# Diagnostic survey of Malagasy *Nesomyrmex* species-groups and revision of *hafahafa* group species via morphology based cluster delimitation protocol

**DOI:** 10.3897/zookeys.526.6037

**Published:** 2015-10-08

**Authors:** Sándor Csősz, Brian L. Fisher

**Affiliations:** 1 Entomology, California Academy of Sciences, 55 Music Concourse Drive, San Francisco, CA 94118, U.S.A.

**Keywords:** Taxonomy, morphometry, species delimitation, exploratory analyses, gap statistic, biogeography

## Abstract

Madagascar and its surrounding islands are among the world’s greatest biodiversity hotspots, harboring predominantly endemic and threatened communities meriting special attention from biodiversity scientists. Building on the considerable efforts in recent years to inventory the Malagasy ant fauna, the myrmicine genus *Nesomyrmex* is reviewed and (1) subdivided into four major groups based on salient morphological features corroborated by numeric morphology: *angulatus*-, *hafahafa*-, *madecassus*- and *sikorai*-groups, and (2) the *hafahafa* species-group endemic to Madagascar is revised. Diversity within *hafahafa* species-group was assessed via hypothesis-free *nest-centroid-clustering* combined with *gap statistic* to assess the number of clusters and to determine the most probable boundaries between them. This combination of methods provides a highly automatized, objective species delineation protocol based on continuous morphometric data. Delimitations of clusters recognized by these exploratory analyses were tested via confirmatory Linear Discriminant Analysis. These results suggest the existence of four morphologically distinct species, *Nesomyrmex capricornis* **sp. n.**, *Nesomyrmex hafahafa* **sp. n.**, *Nesomyrmex medusus* **sp. n.** and *Nesomyrmex spinosus* **sp. n.**; all are described and an identification key for their worker castes using morphometric data is provided. Two members of the newly outlined *hafahafa* species-group, *Nesomyrmex hafahafa* **sp. n.**, *Nesomyrmex medusus* **sp. n.**, are distributed along the southeastern coast Madagascar and occupy rather large ranges, but two other species, *Nesomyrmex capricornis* **sp. n.** and *Nesomyrmex spinosus* **sp. n.**, are only known to occur in small and isolated forest, highlighting the importance of small forest patches for conserving arthropod diversity.

## Introduction

The Malagasy zoogeographical region, i.e. Madagascar and surrounding islands (Bolton 1994), is considered one of the world’s hottest biodiversity hotspots (Myers et al. 2000) and harbors a unique and threatened biota (Ganzhorn et al. 2001). The recently recognized global biodiversity crisis has highlighted the need to explore the flora and fauna of tropical areas, where biodiversity remains largely unexplored, and is enduring the fastest rate of environmental transformation. Thanks to intensive ant systematic research in Madagascar over the last decade (e.g. Fisher 2009, Blaimer and Fisher 2013, Yoshimura and Fisher 2012, Hita-Garcia and Fisher 2014) our knowledge of Malagasy myrmecofauna has increased considerably, supporting earlier assumptions about the extreme species diversity of the region.

However, questions of diversity, rate of endemism, and connections to the African continent for several genera such as Malagasy *Nesomyrmex* have never been the subject of focused research. To date, only four valid *Nesomyrmex* species have been recorded to occur in Madagascar (Mbanyana and Robertson 2008), Based on the recent inventories of Fisher and team, this paper reassesses the *Nesomyrmex* fauna and describes the species from one species group.

A novel approach was used to facilitate species delimitations using multivariate morphometric analyses. Morphological diversity is assessed via NC-clustering (Seifert et al. 2014). This exploratory data analysis technique has proved efficient at pattern recognition within large and complex datasets (Csősz et al. 2014, Guillem et al. 2014, Wachter et al. 2015). The estimation of the optimal number of clusters representing species within a morphological dataset is determined via gap statistic algorithm (Tibshirani et al. 2001). This algorithm helps to find statistically supported number of groups in normally distributed data such as continuous morphometric data based on intra-cluster variance. The combination of NC-clustering and gap statistic offers a highly automated, hypothesis-free protocol producing a statistically calculated goodness of clustering measure that minimizes opportunities for subjective interpretation.

In the present paper, the Malagasy *Nesomyrmex* fauna is subdivided into four clearly delimited species groups diagnosed here and a key to the species groups is provided. The first step of the current project, to inventory the entire Malagasy *Nesomyrmex* fauna, will involve providing a detailed description of the diversity of the *Nesomyrmex hafahafa* species-group. The three pairs of dorsal spines (pronotal spines, propodeal spines and antero-dorsal spines on petiolar node) makes the appearance of this group extremely unique; no similar species group has been found either in the Malagasy region or on the African continent. Multivariate evaluation of morphological data has revealed that the unique-looking *Nesomyrmex hafahafa* species-group comprises four well-outlined clusters, or species, that are endemic to Madagascar. The four new species outlined, *Nesomyrmex capricornis* sp. n., *Nesomyrmex hafahafa* sp. n., *Nesomyrmex medusus* sp. n., and *Nesomyrmex spinosus* sp. n., are described here based on worker caste, and both a key that includes both a numeric identification tool that helps readers to resolve the most problematic cases and a traditional character based key. Distribution maps are also provided. Our research has also revealed that two of the four species, *Nesomyrmex capricornis* sp. n. and *Nesomyrmex spinosus* sp. n., occur in small, highly isolated forests, leaving them at a high risk of extinction from continuing environmental destruction or climatic changes.

## Material and methods

In the present study, 21 continuous morphometric traits were recorded in 177 worker individuals belonging to 100 nest samples collected in the Malagasy region (Table 1). The material is deposited in the California Academy of Sciences (CAS), San Francisco, USA. The full list of non-type material morphometrically examined in this revision is listed in Table 1 with unique specimen identifiers (e.g. CASENT0460666). Designation of type material with detailed label information is given in relevant sections *type material investigated* for each taxon. All images and specimens used in this study are available online on AntWeb (http://www.antweb.org). Images are linked to their specimens via their unique specimen code affixed to each pin (CASENT0002660). Online specimen identifiers follow this format: http://www.antweb.org/specimen/CASENT0002660.

**Table 1.** List of morphometrically investigated samples. Unique CASENT number for pinned samples, locality, geographic coordinates (E, N) in decimal format altitude (ALT) in meters a.s.l., collector’s name, date and number of specimens investigated bearing the given CASENT number are provided. Red row: holotype, yellow row: paratype(s). All samples collected in Toliara administrative region, Madagascar, and deposited at the California Academy of Sciences (CAS).

Species name	CASENT number	Locality	N	E	ALT	Collector	Date	Number of specimens
***capricornis* sp. n. HT**	**CASENT0452741**	**Forêt de Mahavelo, Isantoria River**	**-24,758**	**46,157**	**110 m**	**Fisher-Griswold Arthropod Team**	**1/28/2002**	**1w**
***capricornis* sp. n. PT**	**CASENT0452715**	**Forêt de Mahavelo, Isantoria River**	**-24,758**	**46,157**	**110 m**	**Fisher-Griswold Arthropod Team**	**1/28/2002**	**1w**
***capricornis* sp. n. PT**	**CASENT0452716**	**Forêt de Mahavelo, Isantoria River**	**-24,758**	**46,157**	**110 m**	**Fisher-Griswold Arthropod Team**	**1/28/2002**	**1w**
***capricornis* sp. n. PT**	**CASENT0452738**	**Forêt de Mahavelo, Isantoria River**	**-24,758**	**46,157**	**110 m**	**Fisher-Griswold Arthropod Team**	**1/28/2002**	**2w**
***capricornis* sp. n. PT**	**CASENT0452739**	**Forêt de Mahavelo, Isantoria River**	**-24,758**	**46,157**	**110 m**	**Fisher-Griswold Arthropod Team**	**1/28/2002**	**2w**
***capricornis* sp. n.**	CASENT0443010	Forêt de Mahavelo, Isantoria River	-24,758	46,157	110 m	Fisher-Griswold Arthropod Team	1/28/2002	1w
***capricornis* sp. n.**	CASENT0456949	Parc National d’Andohahela, Forêt de Manantalinjo, 33.6 km 63°ENE Amboasary, 7.6 km 99°E Hazofotsy	-24,817	46,61	150 m	Fisher-Griswold Arthropod Team	1/12/2002	2w
***capricornis* sp. n.**	CASENT0456950	Parc National d’Andohahela, Forêt de Manantalinjo, 33.6 km 63°ENE Amboasary, 7.6 km 99°E Hazofotsy	-24,817	46,61	150 m	Fisher-Griswold Arthropod Team	1/12/2002	2w
***capricornis* sp. n.**	CASENT0452881	Forêt de Mahavelo, Isantoria River	-24,758	46,157	110 m	Fisher-Griswold Arthropod Team	1/28/2002	1w
***capricornis* sp. n.**	CASENT0459109	Parc National d’Andohahela, Forêt de Manantalinjo, 33.6 km 63°ENE Amboasary, 7.6 km 99°E Hazofotsy	-24,817	46,61	150 m	Fisher-Griswold Arthropod Team	1/12/2002	1w
***capricornis* sp. n.**	CASENT0459110	Parc National d’Andohahela, Forêt de Manantalinjo, 33.6 km 63°ENE Amboasary, 7.6 km 99°E Hazofotsy	-24,817	46,61	150 m	Fisher-Griswold Arthropod Team	1/12/2002	1w
***capricornis* sp. n.**	CASENT0456620	Parc National d’Andohahela, Forêt de Manantalinjo, 33.6 km 63°ENE Amboasary, 7.6 km 99°E Hazofotsy	-24,817	46,61	150 m	Fisher-Griswold Arthropod Team	1/12/2002	1w
***capricornis* sp. n.**	CASENT0456621	Parc National d’Andohahela, Forêt de Manantalinjo, 33.6 km 63°ENE Amboasary, 7.6 km 99°E Hazofotsy	-24,817	46,61	150 m	Fisher-Griswold Arthropod Team	1/12/2002	1w
***capricornis* sp. n.**	CASENT0452872	Forêt de Mahavelo, Isantoria River	-24,758	46,157	110 m	Fisher-Griswold Arthropod Team	1/28/2002	2w
***capricornis* sp. n.**	CASENT0452175	Forêt de Mahavelo, Isantoria River	-24,758	46,157	110 m	Fisher-Griswold Arthropod Team	1/28/2002	2w
***capricornis* sp. n.**	CASENT0452871	Forêt de Mahavelo, Isantoria River	-24,758	46,157	110 m	Fisher-Griswold Arthropod Team	1/28/2002	2w
***capricornis* sp. n.**	CASENT0020707	Parc National d’Andohahela, Forêt de Manantalinjo, 33.6 km 63°ENE Amboasary, 7.6 km 99°E Hazofotsy	-24,817	46,61	150 m	Fisher-Griswold Arthropod Team	1/12/2002	1w
***capricornis* sp. n.**	CASENT0079196	Parc National d’Andohahela, Forêt de Manantalinjo, 33.6 km 63°ENE Amboasary, 7.6 km 99°E Hazofotsy	-24,817	46,61	150 m	Fisher-Griswold Arthropod Team	1/12/2002	1w
***capricornis* sp. n.**	CASENT0452754	Forêt de Mahavelo, Isantoria River	-24,758	46,157	110 m	Fisher-Griswold Arthropod Team	1/28/2002	3w
								
***hafahafa* sp. n. HT**	**CASENT0460666**	**Forêt de Tsinjoriaky, 6.2 km 84°E Tsifota**	**-22,802**	**43,421**	**70 m**	**Fisher-Griswold Arthropod Team**	**3/6/2002**	**1w**
***hafahafa* sp. n. PT**	**CASENT0746771**	**Forêt de Tsinjoriaky, 6.2 km 84°E Tsifota**	**-22,802**	**43,421**	**70 m**	**Fisher-Griswold Arthropod Team**	**3/6/2002**	**1w**
***hafahafa* sp. n. PT**	**CASENT0460667**	**Forêt de Tsinjoriaky, 6.2 km 84°E Tsifota**	**-22,802**	**43,421**	**70 m**	**Fisher-Griswold Arthropod Team**	**3/6/2002**	**2w**
***hafahafa* sp. n.**	CASENT0430386	Parc National de Kirindy Mite, 16.3 km 127°SE Belo sur Mer	-20,795	44,147	80 m	Fisher-Griswold Arthropod Team	12/6/2001	2w
***hafahafa* sp. n.**	CASENT0430386	Parc National de Kirindy Mite, 16.3 km 127°SE Belo sur Mer	-20,795	44,147	80 m	Fisher-Griswold Arthropod Team	12/6/2001	2w
***hafahafa* sp. n.**	CASENT0430494	Parc National de Kirindy Mite, 16.3 km 127°SE Belo sur Mer	-20,795	44,147	80 m	Fisher-Griswold Arthropod Team	12/6/2001	2w
***hafahafa* sp. n.**	CASENT0430390	Parc National de Kirindy Mite, 16.3 km 127°SE Belo sur Mer	-20,795	44,147	80 m	Fisher-Griswold Arthropod Team	12/6/2001	2w
***hafahafa* sp. n.**	CASENT0451365	Forêt de Tsinjoriaky, 6.2 km 84°E Tsifota	-22,802	43,421	70 m	Fisher-Griswold Arthropod Team	3/6/2002	2w
***hafahafa* sp. n.**	CASENT0460712	Forêt de Tsinjoriaky, 6.2 km 84°E Tsifota	-22,802	43,421	70 m	Fisher-Griswold Arthropod Team	3/6/2002	2w
***hafahafa* sp. n.**	CASENT0457087	Forêt de Beroboka, 5.9 km 131°SE Ankidranoka	-22,233	43,366	80 m	Fisher-Griswold Arthropod Team	3/12/2002	2w
***hafahafa* sp. n.**	CASENT0439492	Forêt de Beroboka, 5.9 km 131°SE Ankidranoka	-22,233	43,366	80 m	Fisher-Griswold Arthropod Team	3/12/2002	2w
***hafahafa* sp. n.**	CASENT0460679	Forêt de Tsinjoriaky, 6.2 km 84°E Tsifota	-22,802	43,421	70 m	Fisher-Griswold Arthropod Team	3/6/2002	2w
***hafahafa* sp. n.**	CASENT0457090	Forêt de Beroboka, 5.9 km 131°SE Ankidranoka	-22,233	43,366	80 m	Fisher-Griswold Arthropod Team	3/12/2002	2w
***hafahafa* sp. n.**	CASENT0426075	3 km 50°NE Ifaty	-23,15	43,617	60 m	D.O.Burge	10/23/2001	2w
***hafahafa* sp. n.**	CASENT0426077	3 km 50°NE Ifaty	-23,15	43,617	60 m	D.O.Burge	10/23/2001	2w
***hafahafa* sp. n.**	CASENT0059254	Ranobe	-23,045	43,615	20 m	Frontier Wilderness Project	1/26/2004	1w
***hafahafa* sp. n.**	CASENT0446254	Parc National de Kirindy Mite, 16.3 km 127°SE Belo sur Mer	-20,795	44,147	80 m	Fisher-Griswold Arthropod Team	12/6/2001	1w
***hafahafa* sp. n.**	CASENT0066346	Mikea Forest, spiny forest, Tulear Province	-22,913	43,482	37 m	R. Harin’Hala	11/27/2001	1w
***hafahafa* sp. n.**	CASENT0427038	Forêt de Beroboka, 5.9 km 131°SE Ankidranoka	-22,233	43,366	80 m	Fisher-Griswold Arthropod Team	3/12/2002	1w
***hafahafa* sp. n.**	CASENT0447426	Forêt de Tsinjoriaky, 6.2 km 84°E Tsifota	-22,802	43,421	70 m	Fisher-Griswold Arthropod Team	3/6/2002	2w
***hafahafa* sp. n.**	CASENT0447445	Forêt de Tsinjoriaky, 6.2 km 84°E Tsifota	-22,802	43,421	70 m	Fisher-Griswold Arthropod Team	3/6/2002	2w
***hafahafa* sp. n.**	CASENT0447465	Forêt de Tsinjoriaky, 6.2 km 84°E Tsifota	-22,802	43,421	70 m	Fisher-Griswold Arthropod Team	3/6/2002	1w
***hafahafa* sp. n.**	CASENT0127637	48 km ENE Morondava, Kirindy	-20,067	44,65	30 m	B.L.Fisher	4/18/1995	2w
***hafahafa* sp. n.**	CASENT0426078	3 km 50°NE Ifaty	-23,15	43,617	60 m	D.O.Burge	10/23/2001	2w
***hafahafa* sp. n.**	CASENT0430746	Forêt de Tsinjoriaky, 6.2 km 84°E Tsifota	-22,802	43,421	70 m	Fisher-Griswold Arthropod Team	3/6/2002	2w
***hafahafa* sp. n.**	CASENT0459595	Forêt de Tsinjoriaky, 6.2 km 84°E Tsifota	-22,802	43,421	70 m	Fisher-Griswold Arthropod Team	3/6/2002	2w
***hafahafa* sp. n.**	CASENT0004062	Forêt de Tsinjoriaky, 6.2 km 84°E Tsifota	-22,802	43,421	70 m	Fisher-Griswold Arthropod Team	3/6/2002	2w
***hafahafa* sp. n.**	CASENT0457427	Forêt de Beroboka, 5.9 km 131°SE Ankidranoka	-22,233	43,366	80 m	Fisher-Griswold Arthropod Team	3/12/2002	1w
								
***medusus* sp. n. HT**	**CASENT0455428**	**Parc National de Tsimanampetsotsa, Mitoho Cave, 6.4 km 77°ENE Efoetse, 17.4 km 170°S Beheloka**	**-24,047**	**43,753**	**40 m**	**Fisher-Griswold Arthropod Team**	**3/18/2002**	**1w**
***medusus* sp. n. PT**	**CASENT0746770**	**Parc National de Tsimanampetsotsa, Mitoho Cave, 6.4 km 77°ENE Efoetse, 17.4 km 170°S Beheloka**	**-24,047**	**43,753**	**40 m**	**Fisher-Griswold Arthropod Team**	**3/18/2002**	**1w**
***medusus* sp. n.**	CASENT0448719	Mahafaly Plateau, 6.2 km 74°ENE Itampolo	-24,654	43,997	80 m	Fisher-Griswold Arthropod Team	2/21/2002	2w
***medusus* sp. n.**	CASENT0449033	Mahafaly Plateau, 6.2 km 74°ENE Itampolo	-24,654	43,997	80 m	Fisher-Griswold Arthropod Team	2/21/2002	2w
***medusus* sp. n.**	CASENT0449105	Mahafaly Plateau, 6.2 km 74°ENE Itampolo	-24,654	43,997	80 m	Fisher-Griswold Arthropod Team	2/21/2002	2w
***medusus* sp. n.**	CASENT0448791	Mahafaly Plateau, 6.2 km 74°ENE Itampolo	-24,654	43,997	80 m	Fisher-Griswold Arthropod Team	2/21/2002	2w
***medusus* sp. n.**	CASENT0448943	Mahafaly Plateau, 6.2 km 74°ENE Itampolo	-24,654	43,997	80 m	Fisher-Griswold Arthropod Team	37308	2w
***medusus* sp. n.**	CASENT0448945	Mahafaly Plateau, 6.2 km 74°ENE Itampolo	-24,654	43,997	80 m	Fisher-Griswold Arthropod Team	37308	2w
***medusus* sp. n.**	CASENT0451410	Mahafaly Plateau, 6.2 km 74°ENE Itampolo	-24,654	43,997	80 m	Fisher-Griswold Arthropod Team	37308	2w
***medusus* sp. n.**	CASENT0455001	Parc National de Tsimanampetsotsa, Mitoho Cave, 6.4 km 77°ENE Efoetse, 17.4 km 170°S Beheloka	-24,047	43,753	40 m	Fisher-Griswold Arthropod Team	37333	2w
***medusus* sp. n.**	CASENT0448723	Mahafaly Plateau, 6.2 km 74°ENE Itampolo	-24,654	43,997	80 m	Fisher-Griswold Arthropod Team	37308	1w
***medusus* sp. n.**	CASENT0424306	Parc National de Tsimanampetsotsa, Forêt de Bemanateza, 20.7 km 81°E Efoetse, 23.0 km 131°SE Beheloka	-23,992	43,881	90 m	Fisher-Griswold Arthropod Team	37337	1w
***medusus* sp. n.**	CASENT0445085	Parc National de Tsimanampetsotsa, Forêt de Bemanateza, 20.7 km 81°E Efoetse, 23.0 km 131°SE Beheloka	-23,992	43,881	90 m	Fisher-Griswold Arthropod Team	37337	1w
***medusus* sp. n.**	CASENT0444985	Parc National de Tsimanampetsotsa, Forêt de Bemanateza, 20.7 km 81°E Efoetse, 23.0 km 131°SE Beheloka	-23,992	43,881	90 m	Fisher-Griswold Arthropod Team	37337	3w
***medusus* sp. n.**	CASENT0445705	Parc National de Tsimanampetsotsa, Forêt de Bemanateza, 20.7 km 81°E Efoetse, 23.0 km 131°SE Beheloka	-23,992	43,881	90 m	Fisher-Griswold Arthropod Team	3/22/2002	2w
***medusus* sp. n.**	CASENT0445292	Parc National de Tsimanampetsotsa, Forêt de Bemanateza, 20.7 km 81°E Efoetse, 23.0 km 131°SE Beheloka	-23,992	43,881	90 m	Fisher-Griswold Arthropod Team	3/22/2002	2w
***medusus* sp. n.**	CASENT0445591	Parc National de Tsimanampetsotsa, Forêt de Bemanateza, 20.7 km 81°E Efoetse, 23.0 km 131°SE Beheloka	-23,992	43,881	90 m	Fisher-Griswold Arthropod Team	3/22/2002	2w
***medusus* sp. n.**	CASENT0444997	Parc National de Tsimanampetsotsa, Forêt de Bemanateza, 20.7 km 81°E Efoetse, 23.0 km 131°SE Beheloka	-23,992	43,881	90 m	Fisher-Griswold Arthropod Team	3/22/2002	2w
***medusus* sp. n.**	CASENT0427243	Parc National de Tsimanampetsotsa, Forêt de Bemanateza, 20.7 km 81°E Efoetse, 23.0 km 131°SE Beheloka	-23,992	43,881	90 m	Fisher-Griswold Arthropod Team	3/22/2002	2w
***medusus* sp. n.**	CASENT0455177	Parc National de Tsimanampetsotsa, Mitoho Cave, 6.4 km 77°ENE Efoetse, 17.4 km 170°S Beheloka	-24,047	43,753	40 m	Fisher-Griswold Arthropod Team	3/18/2002	2w
***medusus* sp. n.**	CASENT0445705	Parc National de Tsimanampetsotsa, Forêt de Bemanateza, 20.7 km 81°E Efoetse, 23.0 km 131°SE Beheloka	-23,992	43,881	90 m	Fisher-Griswold Arthropod Team	37337	2w
***medusus* sp. n.**	CASENT0445590	Parc National de Tsimanampetsotsa, Forêt de Bemanateza, 20.7 km 81°E Efoetse, 23.0 km 131°SE Beheloka	-23,992	43,881	90 m	Fisher-Griswold Arthropod Team	3/22/2002	2w
***medusus* sp. n.**	CASENT0445291	Parc National de Tsimanampetsotsa, Forêt de Bemanateza, 20.7 km 81°E Efoetse, 23.0 km 131°SE Beheloka	-23,992	43,881	90 m	Fisher-Griswold Arthropod Team	3/22/2002	4w
***medusus* sp. n.**	CASENT0004002	Mahafaly Plateau, 6.2 km 74°ENE Itampolo	-24,654	43,997	80 m	Fisher-Griswold Arthropod Team	2/21/2002	2w
***medusus* sp. n.**	CASENT0477179	Parc National de Tsimanampetsotsa, 6.7 km 130°SE Efoetse, 23.0 km 175°S Beheloka	-24,101	43,76	25 m	Fisher-Griswold Arthropod Team	3/18/2002	1w
***medusus* sp. n.**	CASENT0477180	Parc National de Tsimanampetsotsa, 6.7 km 130°SE Efoetse, 23.0 km 175°S Beheloka	-24,101	43,76	25 m	Fisher-Griswold Arthropod Team	3/18/2002	1w
***medusus* sp. n.**	CASENT0455436	Parc National de Tsimanampetsotsa, Mitoho Cave, 6.4 km 77°ENE Efoetse, 17.4 km 170°S Beheloka	-24,047	43,753	40 m	Fisher-Griswold Arthropod Team	3/18/2002	2w
***medusus* sp. n.**	CASENT0454945	Parc National de Tsimanampetsotsa, Mitoho Cave, 6.4 km 77°ENE Efoetse, 17.4 km 170°S Beheloka	-24,047	43,753	40 m	Fisher-Griswold Arthropod Team	3/18/2002	2w
***medusus* sp. n.**	CASENT0454890	Parc National de Tsimanampetsotsa, Mitoho Cave, 6.4 km 77°ENE Efoetse, 17.4 km 170°S Beheloka	-24,047	43,753	40 m	Fisher-Griswold Arthropod Team	3/18/2002	2w
***medusus* sp. n.**	CASENT0455002	Parc National de Tsimanampetsotsa, Mitoho Cave, 6.4 km 77°ENE Efoetse, 17.4 km 170°S Beheloka	-24,047	43,753	40 m	Fisher-Griswold Arthropod Team	3/18/2002	2w
***spinosus* sp. n. *HT***	**CASENT0443515**	**Réserve Privé Berenty, Forêt d’Anjapolo, 21.4 km 325°NW Amboasary**	**-24,930**	**46,210**	**65 m**	**Fisher-Griswold Arthropod Team**	**2/7/2002**	**1w**
***spinosus* sp. n. *PT***	**CASENT0443515**	**Réserve Privé Berenty, Forêt d’Anjapolo, 21.4 km 325°NW Amboasary**	**-24,930**	**46,210**	**65 m**	**Fisher-Griswold Arthropod Team**	**2/7/2002**	**1w**
***spinosus* sp. n. *PT***	**CASENT0443531**	**Réserve Privé Berenty, Forêt d’Anjapolo, 21.4 km 325°NW Amboasary**	**-24,930**	**46,210**	**65 m**	**Fisher-Griswold Arthropod Team**	**2/7/2002**	**1w**
***spinosus* sp. n.**	CASENT0454095	Parc National d’Andohahela, Forêt d’Ambohibory, 1.7 km 61°ENE Tsimelahy, 36.1 km 308°NW Tolagnaro	-24,93	46,646	300 m	Fisher-Griswold Arthropod Team	1/16/2002	3w
***spinosus* sp. n.**	CASENT0454237	Parc National d’Andohahela, Forêt d’Ambohibory, 1.7 km 61°ENE Tsimelahy, 36.1 km 308°NW Tolagnaro	-24,93	46,646	300 m	Fisher-Griswold Arthropod Team	1/16/2002	2w
***spinosus* sp. n.**	CASENT0454238	Parc National d’Andohahela, Forêt d’Ambohibory, 1.7 km 61°ENE Tsimelahy, 36.1 km 308°NW Tolagnaro	-24,93	46,646	300 m	Fisher-Griswold Arthropod Team	1/16/2002	2w
***spinosus* sp. n.**	CASENT0454100	Parc National d’Andohahela, Forêt d’Ambohibory, 1.7 km 61°ENE Tsimelahy, 36.1 km 308°NW Tolagnaro	-24,93	46,646	300 m	Fisher-Griswold Arthropod Team	1/16/2002	2w
***spinosus* sp. n.**	CASENT0001365	Parc National d’Andohahela, Forêt d’Ambohibory, 1.7 km 61°ENE Tsimelahy, 36.1 km 308°NW Tolagnaro	-24,93	46,646	300 m	Fisher-Griswold Arthropod Team	1/16/2002	1w
***spinosus* sp. n.**	CASENT0001366	Parc National d’Andohahela, Forêt d’Ambohibory, 1.7 km 61°ENE Tsimelahy, 36.1 km 308°NW Tolagnaro	-24,93	46,646	300 m	Fisher-Griswold Arthropod Team	1/16/2002	1w
***spinosus* sp. n.**	CASENT0003947	Réserve Privé Berenty, Forêt d’Anjapolo, 21.4 km 325°NW Amboasary	-24,930	46,210	65 m	Fisher-Griswold Arthropod Team	2/7/2002	2w
***spinosus* sp. n.**	CASENT0001369	Parc National d’Andohahela, Forêt d’Ambohibory, 1.7 km 61°ENE Tsimelahy, 36.1 km 308°NW Tolagnaro	-24,93	46,646	300 m	Fisher-Griswold Arthropod Team	1/16/2002	2w
***spinosus* sp. n.**	CASENT0454236	Parc National d’Andohahela, Forêt d’Ambohibory, 1.7 km 61°ENE Tsimelahy, 36.1 km 308°NW Tolagnaro	-24,93	46,646	300 m	Fisher-Griswold Arthropod Team	1/16/2002	2w
***spinosus* sp. n.**	CASENT0057339	Réserve Privé Berenty, Forêt d’Anjapolo, 21.4 km 325°NW Amboasary	-24,930	46,210	65 m	B.L.Fisher	4/16/2005	1w
***spinosus* sp. n.**	CASENT0454094	Parc National d’Andohahela, Forêt d’Ambohibory, 1.7 km 61°ENE Tsimelahy, 36.1 km 308°NW Tolagnaro	-24,93	46,646	300 m	Fisher-Griswold Arthropod Team	1/16/2002	2w
***spinosus* sp. n.**	CASENT0443504	Réserve Privé Berenty, Forêt d’Anjapolo, 21.4 km 325°NW Amboasary	-24,930	46,210	65 m	Fisher-Griswold Arthropod Team	2/7/2002	1w
***spinosus* sp. n.**	CASENT0443512	Réserve Privé Berenty, Forêt d’Anjapolo, 21.4 km 325°NW Amboasary	-24,930	46,210	65 m	Fisher-Griswold Arthropod Team	2/7/2002	2w
***spinosus* sp. n.**	CASENT0443601	Réserve Privé Berenty, Forêt d’Anjapolo, 21.4 km 325°NW Amboasary	-24,930	46,210	65 m	Fisher-Griswold Arthropod Team	2/7/2002	2w
***spinosus* sp. n.**	CASENT0442542	Réserve Privé Berenty, Forêt d’Anjapolo, 21.4 km 325°NW Amboasary	-24,930	46,210	65 m	Fisher-Griswold Arthropod Team	2/7/2002	1w
***spinosus* sp. n.**	CASENT0443593	Réserve Privé Berenty, Forêt d’Anjapolo, 21.4 km 325°NW Amboasary	-24,930	46,210	65 m	Fisher-Griswold Arthropod Team	2/7/2002	2w
***spinosus* sp. n.**	CASENT0443501	Réserve Privé Berenty, Forêt d’Anjapolo, 21.4 km 325°NW Amboasary	-24,930	46,210	65 m	Fisher-Griswold Arthropod Team	2/7/2002	1w
***spinosus* sp. n.**	CASENT0443502	Réserve Privé Berenty, Forêt d’Anjapolo, 21.4 km 325°NW Amboasary	-24,930	46,210	65 m	Fisher-Griswold Arthropod Team	2/7/2002	1w
***spinosus* sp. n.**	CASENT0442540	Réserve Privé Berenty, Forêt d’Anjapolo, 21.4 km 325°NW Amboasary	-24,930	46,210	65 m	Fisher-Griswold Arthropod Team	2/7/2002	1w
***spinosus* sp. n.**	CASENT0442541	Réserve Privé Berenty, Forêt d’Anjapolo, 21.4 km 325°NW Amboasary	-24,930	46,210	65 m	Fisher-Griswold Arthropod Team	2/7/2002	1w
***spinosus* sp. n.**	CASENT0443539	Réserve Privé Berenty, Forêt d’Anjapolo, 21.4 km 325°NW Amboasary	-24,930	46,210	65 m	Fisher-Griswold Arthropod Team	2/7/2002	2w
***spinosus* sp. n.**	CASENT0443544	Réserve Privé Berenty, Forêt d’Anjapolo, 21.4 km 325°NW Amboasary	-24,930	46,210	65 m	Fisher-Griswold Arthropod Team	2/7/2002	2w
***spinosus* sp. n.**	CASENT0443540	Réserve Privé Berenty, Forêt d’Anjapolo, 21.4 km 325°NW Amboasary	-24,930	46,210	65 m	Fisher-Griswold Arthropod Team	2/7/2002	2w
***spinosus* sp. n.**	CASENT0001469	Parc National d’Andohahela, Forêt d’Ambohibory, 1.7 km 61°ENE Tsimelahy, 36.1 km 308°NW Tolagnaro	-24,93	46,646	300 m	Fisher-Griswold Arthropod Team	1/16/2002	2w
***spinosus* sp. n.**	CASENT0443605	Réserve Privé Berenty, Forêt d’Anjapolo, 21.4 km 325°NW Amboasary	-24,930	46,210	65 m	Fisher-Griswold Arthropod Team	2/7/2002	3w
***spinosus* sp. n.**	CASENT0108875	Anosy Region, Distric of Amboasary,58Km SW of Fort Dauphin, 08Km NW of Amboasary, Berenty Special Reserve	-25,021	46,306	36 m	Mike, Rin’ha	11/30/2003	1w

Digital color montage images were created using a JVC KY-F75 digital camera and Syncroscopy Auto-Montage software (version 5.0), or a Leica DFC 425 camera in combination with the Leica Application Suite software (version 3.8). Distribution maps were generated by using QGIS 2.4.0 software (QGIS Development Team 2014).

**Figures 1–6.**
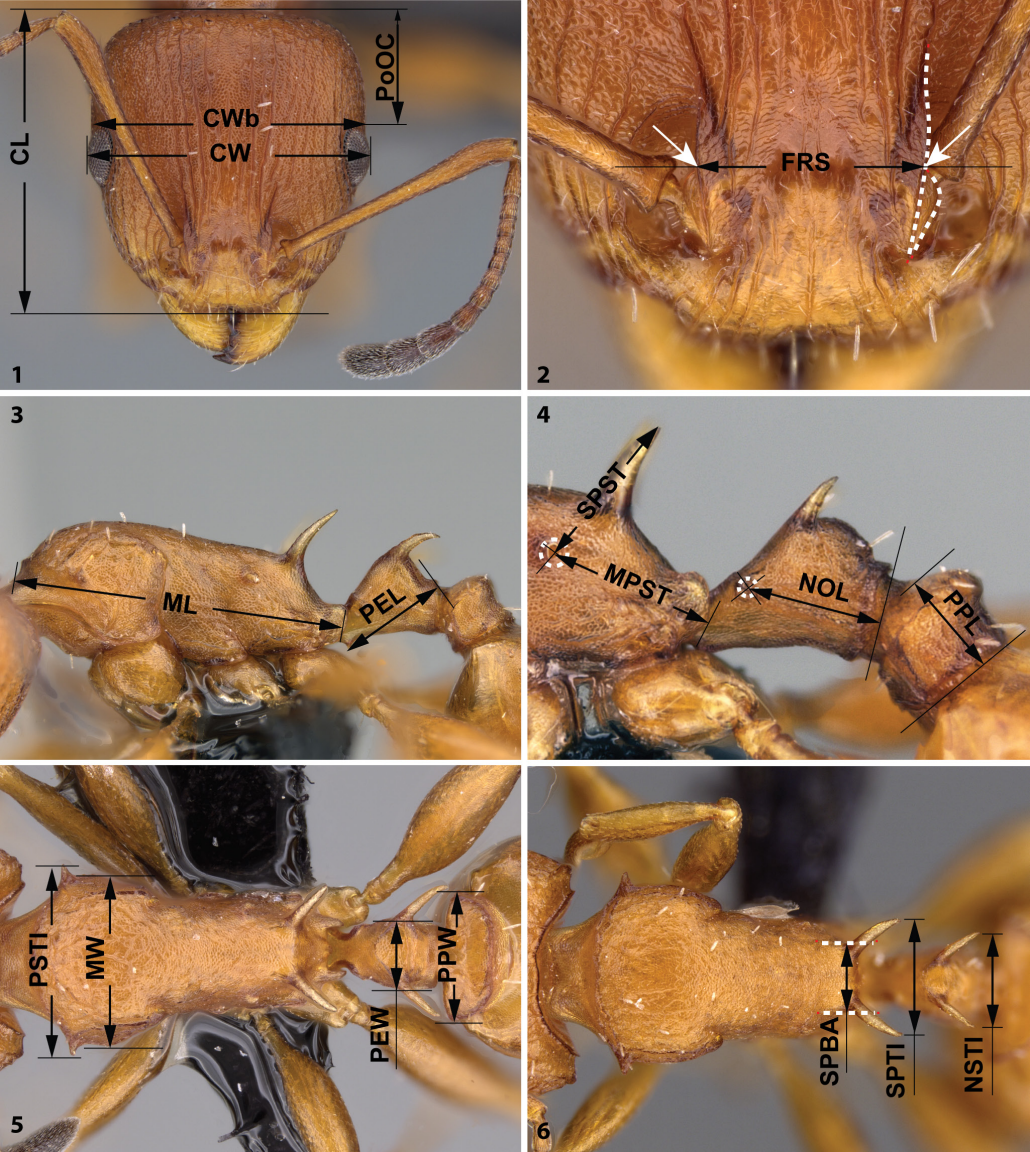
Measurement lines for metric characters. Head in dorsal view (**1**) with measurement lines for CL, CW, CWB and PoOC; frontal region of the head dorsum (**2**) with measurement lines for FRS; dorsal view of mesosoma (**3**) with measurement lines for NSTI, SPBA and SPTI; dorsal view of mesosoma (**4**) with measurement lines for MW, PSTI, PEW and PPW; lateral view of mesosoma (**5**) with measurement lines for ML and PEL; lateral view of mesosoma petiole and postpetiole (**6**) with measurement lines for MPST, NOL, PPL and SPST.

The measurements were taken with a Leica MZ 12.5 stereomicroscope equipped with an ocular micrometer at a magnification of 100×. Measurements and indices are presented as arithmetic means with minimum and maximum values in parentheses. Body size dimensions are expressed in µm. Due to the abundance of worker individuals in contrast to the limited number of queen and male specimens available the present revision is based on worker caste only. Worker-based revision is further facilitated by the fact that name-bearing type specimens of the vast majority of existing ant taxa were designated from worker caste. All measurements were made by the first author. For the definition of morphometric characters, earlier protocols (Schlick-Steiner et al. 2006, Seifert 2006, Seifert and Csősz 2015) were considered. Explanations and abbreviations for measured characters are as follows:

CL Maximum cephalic length in median line. The head must be carefully tilted to the position providing the true maximum. Excavations of hind vertex and/or clypeus reduce CL (Fig. 1).

CW Maximum width of the head including compound eyes (Fig. 1).

CWb Maximum width of head capsule without the compound eyes. Measured just posterior of the eyes (Fig. 1).

Cdep Antero-median clypeal depression. Maximum depth of the median clypeal depression on its anterior contour line as it appears in fronto-dorsal view.

EL Maximum diameter of the compound eye.

FRS Frontal carina distance. Distance of the frontal carinae immediately caudal of the posterior intersection points between frontal carinae and the torular lamellae. If these dorsal lamellae do not laterally surpass the frontal carinae, the deepest point of scape corner pits may be taken as reference line. These pits take up the inner corner of scape base when the scape is fully directed caudally and produces a dark triangular shadow in the lateral frontal lobes immediately posterior to the dorsal lamellae of the scape joint capsule (Fig. 2).

ML
**(Weber length)** Mesosoma length from caudalmost point of propodeal lobe to transition point between anterior pronotal slope and anterior pronotal shield (preferentially measured in lateral view; if the transition point is not well defined, use dorsal view and take the centre of the dark-shaded borderline between pronotal slope and pronotal shield as anterior reference point). In gynes: length from caudalmost point of propodeal lobe to the most distant point of steep anterior pronotal face (Fig. 3).

MPST Maximum distance from the center of the propodeal spiracle to the posteroventral corner of the ventrolateral margin of the metapleuron (Fig. 4).

MW Mesosoma width. In workers MW is defined as the longest width of the pronotum in dorsal view excluding the pronotal spines (Fig. 5).

NOL Length of the petiolar node. Measured in lateral view from the centre of petiolar spiracle to dorso-caudal corner of caudal cylinder. Do not erroneously take as the reference point the dorso-caudal corner of the helcium, which is sometimes visible (Fig. 4).

NSTI Apical distance of the anterodorsal spines on the petiolar node in dorsal view; if spine tips are rounded or thick take the centers of spine tips as reference points (Fig. 6).

PEL Diagonal petiolar length in lateral view; measured from anterior corner of subpetiolar process to dorso-caudal corner of caudal cylinder (Fig. 3).

PEW Maximum width of petiole in dorsal view. Nodal spines are not considered (Fig. 5).

PoOC Postocular distance. Use a cross-scaled ocular micrometer and adjust the head to the measuring position of CL. Caudal measuring point: median occipital margin; frontal measuring point: median head at the level of the posterior eye margin (Fig. 1).

PPL Postpetiole length. The longest anatomical line that is perpendicular to the posterior margin of the postpetiole and is between the posterior postpetiolar margin and the anterior postpetiolar margin (Fig. 4).

PPW Postpetiole width. Maximum width of postpetiole in dorsal view (Fig. 5).

PSTI Apical distance of pronotal spines in dorsal view; if spine tips are rounded or thick take the centers of spine tips as reference points (Fig. 5).

SL Scape length. Maximum straight line scape length excluding the articular condyle.

SPBA Minimum propodeal spine distance. The smallest distance of the lateral margins of the propodeal spines at their base. This should be measured in dorsofrontal view, since the wider parts of the ventral propodeum do not interfere with the measurement in this position. If the lateral margins of propodeal spines diverge continuously from the tip to the base, a smallest distance at base is not defined. In this case, SPBA is measured at the level of the bottom of the interspinal meniscus (Fig. 6).

SPST Propodeal spine length. Distance between the centre of propodeal spiracle and spine tip. The spiracle centre refers to the midpoint defined by the outer cuticular ring but not to the centre of real spiracle opening that may be positioned eccentrically (Fig. 4).

SPTI Apical propodeal spine distance. The distance of propodeal spine tips in dorsal view; if spine tips are rounded or truncated, the centres of spine tips are taken as reference points (Fig. 6).

Taxonomic nomenclature, OTU concepts and natural language (NL) phenotypes were compiled in mx (http://purl.org/NET/mx-database). Taxonomic history and descriptions of taxonomic treatments were rendered from this software. Hymenoptera-specific terminology of morphological statements used in descriptions, identification key, and diagnoses are mapped to classes in phenotype-relevant ontologies (Hymenoptera Anatomy Ontology (HAO) (Yoder et al. 2010) via a URI table (Table 2); see Seltmann et al. (2012), Mikó et al. (2014) for more information about this approach.

**Table 2.** URI table for morphometric characters and Hymenoptera-specific terminology of morphological statements used in descriptions, identification key, and diagnoses are mapped to classes in phenotype-relevant ontologies.

Abbr.	Label	Class genus differentia definition	Comments	uri
CL	maximum cephalic length in median view	The median anatomical line that extends between the posterior margin of the cranium and the distal margin of the clypeus in frontal view.	The maximum cephalic length in median view is not equivalent to the maximum cephalic size that extends between the posterior cranial margin and the distal clypeal line. The head must be carefully tilted to the position with the true maximum. Excavations of hind vertex and/or clypeus reduce CL (Fig. 1A).	http://purl.obolibrary.org/obo/HAO_0002331
CW	head width	The anatomical line that is the longest horizontal diameter of the cranium in frontal view.	The head width is the largest distance between the lateral margins of the compound eyes measured in frontal view (Fig. 1A).	http://purl.obolibrary.org/obo/HAO_0002268
CWb	dorsal head width	The anatomical line between the intersections of the cranium contour line and dorsal head line in frontal view.	The dorsal head width is the maximum width of head capsule without the compound eyes that is measured just posterior of the eyes in frontal view (Fig. 1A).	http://purl.obolibrary.org/obo/HAO_0002314
Cdep	median clypeal notch depth	The anatomical line that is between the distal clypeal line and the proximalmost point of the distal clypeal notch in frontal view.		http://purl.obolibrary.org/obo/HAO_0002333
EL	maximum diameter of compound eye	The longest diameter of the eye.		http://purl.obolibrary.org/obo/HAO_0002326
FRS	frontal carina line	The transverse torular line that extends between the frontal carinae.	Distance of the frontal carinae immediately caudal of the posterior intersection points between frontal carinae and the torular lamellae. If these dorsal lamellae do not laterally surpass the frontal carinae, the deepest point of scape corner pits may be taken as reference line. These pits take up the inner corner of scape base when the scape is fully switched caudad and produce a dark triangular shadow in the lateral frontal lobes immediately posterior of the dorsal lamellae of scape joint capsule (Fig. 1B).	http://purl.obolibrary.org/obo/HAO_0002323
ML	Weber length	The anatomical line that connects the global minima of the contour line of the pronotal slope in lateral view when the specimen is rotated until the contour line becames as symmetric as possible and the posteriormost point of the propodeal lobe.	Preferentially measured in lateral view; if the transition point is not well defined, use dorsal view and take the centre of the dark-shaded borderline between pronotal slope and pronotal shield as anterior reference point. In gynes: length from distalmost point of propodeal lobe to the most distant point of steep anterior pronotal face (Fig. 1E).	http://purl.obolibrary.org/obo/HAO_0002309
MPST	maximum spiracle distance of propodeum	The anatomical line that connects the center of the propodeal spiracle with the posteriormost point of the propodeal lobe in lateral view.	Maximum distance from the center of the propodeal stigma to the anterioventral corner of the ventrolateral margin of the metapleuron (Fig. 1F).	http://purl.obolibrary.org/obo/HAO_0002334
MW	mesosoma width	The longest width of the pronotum in dorsal view.	Mesosoma width. In workers MW is defined as the longest width of the pronotum in dorsal view excluding the pronotal spines (Fig. 1D).	http://purl.obolibrary.org/obo/HAO_0002335
NOL	length of petiolar node	The anatomical line that is the shortest between the center of the petiolar spiracle and the posterior margin of the petiole in lateral view.	Length of the petiolar node. Measured in lateral view from the centre of petiolar spiracle to posterodorsal corner of caudal cylinder. Do not erroneously take as reference point the dorso-caudal corner of the helcium, which is sometimes visible (Fig. 1F).	http://purl.obolibrary.org/obo/HAO_0002336
NOH	maximum height of petiolar node	The anatomical line that is the longest between the dorsal margin of the petiole and the posterior petiolar distance and perpendicular to the posterior petiolar distance.		http://purl.obolibrary.org/obo/HAO_0002327
NSTI	apical petiolar spine distance	The anatomical line between the distal ends of the anterodorsal spines of the petiolar node.	If spine tips are rounded or thick take the centers of spine tips as reference points (Fig. 1C).	http://purl.obolibrary.org/obo/HAO_0002338
PEH	maximum petiole height	The anatomical line that is the longest between the ventral margin of the petiole and the dorsal margin of the petiole and is perpendicular to the ventral margin of the petiole in lateral view.		http://purl.obolibrary.org/obo/HAO_0002328
PEL	diagonal petiolar length	The anatomical line that extends between the distalmost point of the subpetiolar process and the global minima of the contour line of the dorsal region of the posterior petiolar constriction in lateral view when the specimen is rotated until the contour line became as symmetric as possible.	Fig. 1E.	http://purl.obolibrary.org/obo/HAO_0002317
PEW	petiole width	The maximum width of the petiole in dorsal view.	Anterodorsal spines of the petiolar node are not considered (Fig. 1D).	http://purl.obolibrary.org/obo/HAO_0002339
PoOC	postocular distance	The median anatomical line of the cranium that is the longest between the dorsal margin of the cranium and the dorsal head width.	Use a cross-scaled ocular micrometer and adjust the head to the measuring position of CL. Caudal measuring point: median occipital margin; frontal measuring point: median head at the level of the posterior eye margin (Fig. 1A).	http://purl.obolibrary.org/obo/HAO_0002340
PPL	postpetiole length	The longest anatomical line that is perpendicular to the posterior margin of the postpetiole in lateral view and is between the posterior postpetiolar margin and the anterior postpetiolar margin.	Fig. 1F	http://purl.obolibrary.org/obo/HAO_0002341
PPW	postpetiole width	The maximum width of the postpetiole in dorsal view.	Fig. 1D	http://purl.obolibrary.org/obo/HAO_0002342
PSTI	apical distance of pronotal spines	The anatomical line between the distal ends of the pronotal spines.	If spine tips are rounded or thick take the centers of spine tips as reference points (Fig. 1D).	http://purl.obolibrary.org/obo/HAO_0002345
SL	scape length	The proximodistal anatomical line of the scapal area distal to the radicle.	Maximum straight line scape length excluding the radicle (Fig. 1A).	http://purl.obolibrary.org/obo/HAO_0002346
SPBA	minimum spine distance	The shortest anatomical line between the lateral margins of the propodeal spines.	This should be measured in anterodorsal view, since the wider parts of the ventral propodeum do not interfere with the measurement in this position. If the lateral margins of spines diverge continuously from the tip to the base, a smallest distance at base is not defined. In this case, SPBA is measured at the level of the bottom of the interspinal meniscus (Fig. 1C).	http://purl.obolibrary.org/obo/HAO_0002347
SPST	spine length	The anatomical line between the center of the propodeal spiracle and the distal end of the propodeal spine.	Spine length. Distance between the centre of propodeal stigma and spine tip. The stigma centre refers to the midpoint defined by the outer cuticular ring but not to the centre of real stigma opening that may be positioned eccentrically (Fig. 1F).	http://purl.obolibrary.org/obo/HAO_0002348
SPTI	apical spine distance	The anatomical line between the distal ends of the propodeal spines.	If spine tips are rounded or truncated, the centres of spine tips are taken as reference points (Fig. 1C).	http://purl.obolibrary.org/obo/HAO_0002319
	anterior pronotal slope	The concave area anteriorly on the mesosoma that accommodates the posterior area of the cranium.		http://purl.obolibrary.org/obo/HAO_0002311
	anterior setal pit	The anteriormost setal pit on the dorsal side of the petiole.		http://purl.obolibrary.org/obo/HAO_0002312
	caudal cylinder	The petiolar area posterior to the posterior petiolar constriction.		http://purl.obolibrary.org/obo/HAO_0002318
	cranial scrobe of the pronotum	The scrobe on the pronotum that accommodates the posterior surface of the cranium.		http://purl.obolibrary.org/obo/HAO_0002343
	distal clypeal line	The anatomical line that is perpendicular to the median anatomical line and is the tangent at the distalmost point(s) of the clypeus in frontal view.		http://purl.obolibrary.org/obo/HAO_0002316
	dorsal head line	The anatomical line between the posteriormost (dorsalmost) points of compound eyes in frontal view.		http://purl.obolibrary.org/obo/HAO_0002315
	dorsal petiolar scrobe	The scrobe that is dorsal to the propodeal foramen and accommodates the proximodorsal area of the petiole.		http://purl.obolibrary.org/obo/HAO_0002313
	external area of the scape	The area of the scape that faces away from the cranial surface in fully caudal scape position.		http://purl.obolibrary.org/obo/HAO_0002320
	eye	The compound organ that is composed of ommatidia.		http://purl.obolibrary.org/obo/HAO_0000217
	facial area of the scape	The area of the scape that faces the cranium surface when the scape is in fully flexed position.		http://purl.obolibrary.org/obo/HAO_0002321
	frontal carina	The carina that extends along the lateral margin of the intertorular area (median margin of the antennal foramen) towards the vertex.		http://purl.obolibrary.org/obo/HAO_0001533
	frontal carina line	The transverse torular line that extends between the frontal carinae.		http://purl.obolibrary.org/obo/HAO_0002323
	lateral carina of clypeus	The carina that extends between the ventral (anterior) margin of the antennal foramen to the apical clypeal margin.		http://purl.obolibrary.org/obo/HAO_0002324
	margin	The line that delimits the periphery of an area.		http://purl.obolibrary.org/obo/HAO_0000510
	median clypeal notch	The median notch that is on the distal clypeal margin.		http://purl.obolibrary.org/obo/HAO_0002332
	mesosoma	The anatomical cluster that is composed of the prothorax, mesothorax and the metapectal-propodeal complex.		http://purl.obolibrary.org/obo/HAO_0000576
	Weber length	The anatomical line that connects the global minima of the contour line of the pronotal slope in lateral view when the specimen is rotated until the contour line becames as symmetric as possible and the posteriormost point of the propodeal lobe.		http://purl.obolibrary.org/obo/HAO_0002309
	petiolar scrobe	The scrobe that is located ventrally of the propodeal foramen and accommodates the proximal area of the petiole.		http://purl.obolibrary.org/obo/HAO_0002265
	pronotal spine	The spine that is located at the dorsolateral edge of the cranial scrobe of the pronotum.		http://purl.obolibrary.org/obo/HAO_0002344
	pronotum	The notum that is located in the prothorax.		http://purl.obolibrary.org/obo/HAO_0000853
	scape	The antennal segment that is proximal to the pedicel and is connected to the head via the radicle.		http://purl.obolibrary.org/obo/HAO_0000908
	scrobe	The area that is impressed and is for the reception or concealment of another sclerite.		http://purl.obolibrary.org/obo/HAO_0000912
	setal angle	The angle of the proximodistal axis of the seta to the contour line of the bodypart where the seta is located.		http://purl.obolibrary.org/obo/HAO_0002330
	setal line	The row that is composed of setae.		http://purl.obolibrary.org/obo/HAO_0000903
	setal pit	The impression with a centered sensillum trichodeum.		http://purl.obolibrary.org/obo/HAO_0001958
	spine	The process that lacks non-sclerotised ring at the base.		http://purl.obolibrary.org/obo/HAO_0000949
	spiracle	The anatomical cluster that is composed of the distal end of the trachea and the margin of the sclerite or conjunctiva surrounding the spiracular opening.		http://purl.obolibrary.org/obo/HAO_0000950
	transverse torular line	The anatomical line that is tangential to the posteriormost points of the antennal rims.		http://purl.obolibrary.org/obo/HAO_0002322
	width	A 1-D extent quality which is equal to the distance from one side of an object to another side which is opposite.		http://purl.obolibrary.org/obo/HAO_0002308

In verbal descriptions of taxa based on external morphological traits, recent taxonomic papers (Csősz et al. 2014, Seifert and Csősz 2015) were considered. Definitions of surface sculpturing are linked to Harris (1979). Body size is given in µm, means of morphometric ratios as well as minimum and maximum values are given in parentheses with up to three digits. Estimated inclination of pilosity and cuticular spines is given in degrees. Definitions of species-groups as well as descriptions of species are surveyed in alphabetic order.

### Statistical analyses of continuous morphometric data

*Hypothesis formation by exploratory analyses.* Our hypothesis of the number of clusters and classification of samples was formulated by an exploratory data analysis technique, NC-clustering (Seifert et al. 2014) using continuous morphometric data. NC-clustering searches for discontinuities in data, sorting all similar cases into the same cluster by transforming morphological differences between nest samples into a distance matrix in a linear discriminant space. The linear discriminant scores for each nest sample are displayed in a dendrogram within Euclidean space via UPGMA (Unweighted Pair Group Method with Arithmetic Mean) distance method. This method is able to tackle large datasets with high dimensionality (Csősz et al. 2014, Guillem et al. 2014, Wachter et al. 2015), providing readily inferable patterns even for a high number of clusters. A bootstrap version of cluster analysis was applied to evaluate how consistently the same clusters appear with a sub-sampled dataset by running 1000 iterations (method = “average”, method.dist = “euclidean”, nboot = 1000) using package *pvclust* (Suzuki and Shimodaira 2014). Package *pvclust* returns two type of p values: the Approximately Unbiased P-value (AU) is computed by multiscale bootstrap resampling, and the raw Bootstrap Probabilities (BP) that is calculated before statistical adjustments by normal bootstrap resampling.

The optimal number of clusters was determined via gap statistic using gap criterion introduced by Tibshirani et al. (2001). The gap statistic is a standard method for determining the number of clusters in a set of data (Mohajer et al. 2010). It clusters the observed data, varying the number of clusters and computes the corresponding within-cluster dispersion (i.e. the sum of the squared distances between the observations and the center of the cluster). For each number of clusters the gap statistic compares the standardized within-cluster dispersion to its expectation under an appropriate null reference distribution (i.e. each observation is assumed to fall in a single cluster). The optimal number of clusters is the value for which the observed within-cluster dispersion falls the farthest below this reference curve (Tibshirani et al. 2001).

Statistical computing was done in R (R Core Team 2014). NC-clustering was done via package *cluster* (Maechler et al. 2014), *MASS* (Venables and Ripley 2002). Gap statistic and partitioning of samples was calculated based on recursive thresholding via the *clusterGenomics* package (Nilsen and Lingjaerde 2013) using functions ‘gap’ (with optional arguments Kmax=10, B=100, nstart=20) and ‘part’ (Kmax=10, minSize=5, Kmax.rec=5, B=100).

*Hypothesis testing by confirmatory LDA.* To increase the reliability of species delimitation, hypotheses on clusters and classifications of cases via two exploratory processes were tested by a confirmative LDA. Classification hypotheses were imposed for all samples congruently classified by exploratory methods while wild-card settings (i.e. no prior hypothesis imposed on its classification) were given to samples that were incongruently classified by the two methods. The confirmative LDA was run as an iterative process to achieve the lowest number of characters necessary to achieve the desired level (>97%) of classification success (Seifert 2014).

## Results

### Synopsis of Malagasy *Nesomyrmex* species

angulatus group

***angulatus*** (Mayr, 1862)

= *angulatus ilgii* (Forel, 1894)

= *latinodis* (Mayr, 1895)

= *angulatus concolor* (Santschi, 1914)

hafahafa group

***capricornis*** Csősz & Fisher,sp. n.

***hafahafa*** Csősz & Fisher,sp. n.

***medusus*** Csősz & Fisher,sp. n.

***spinosus*** Csősz & Fisher,sp. n.

madecassus group

***gibber*** (Donisthorpe, 1946)

***madecassus*** (Forel, 1892)

sikorai group

***retusispinosus*** (Forel, 1892)

***sikorai*** (Emery, 1896)

### I. Definitions and diagnoses of groups

#### Key to species-groups

**Table:** 

1	Anterodorsal spines on petiolar node present (Fig. 7)	***hafahafa* group**
–	Anterodorsal spines on petiolar node absent (Figs 8–11)	**2**
2	Petiolar node globular in dorsal view (Fig. 8), postocular distance vs. petiole width (PoOc/PEW): 0.887 [0.723, 1.167]	***angulatus* group**
–	Petiolar node long and narrow in dorsal view, sides are nearly parallel (Fig. 9). Postocular distance vs. petiole width (PoOc/PEW): (*sikorai*-group) 1.415 [1.198, 1.676], (*madecassus*-group) 1.610 [1.210, 2.090]	**3**
3	Petiolar node in lateral view lower, (MPST/NOH): 3.541 [2.714, 5.625], propodeal spines very short to absent, mesopropodeal depression absent to shallow (Fig. 10)	***madecassus* group**
–	Petiolar node in lateral view higher, (MPST/NOH): 2.409 [1.885, 2.869], propodeal spines moderately long, always present, mesopropodeal depression conspicuous, deep (Fig. 11)	***sikorai* group**

**Figures 7–11.**
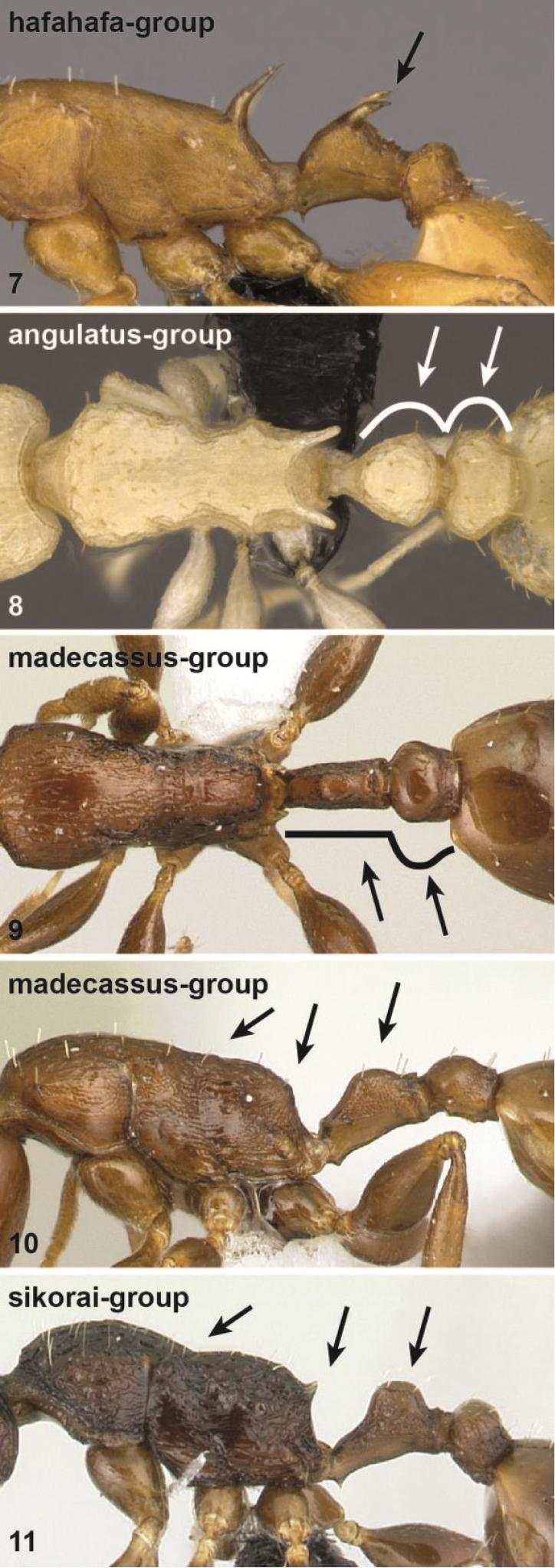
Diagnostic characters for workers of all species-groups outlined in this paper. Lateral view of mesosoma, petiole and postpetiole of a member of the *hafahafa* species-group (**7**), dorsal view of mesosoma, petiole and postpetiole of *angulatus* species-group (**8**), dorsal view of mesosoma, petiole and postpetiole of *madecassus* species-group (**9**), lateral view of mesosoma, petiole and postpetiole of *madecassus* species-group (**10**), lateral view of mesosoma, petiole and postpetiole of *sikorai* species-group (**11**). For details see main text.

#### *angulatus* species-group

Pronotal spines present or absent. Anterodorsal spines on petiolar node absent. Propodeal spines short to long and acute. Vertex ground sculpture areolate. Main sculpture on vertex not defined. Metanotal depression present or absent. Median clypeal notch present or absent. Median clypeal notch shape/depth: 0–23 µm. Antennomere count: 12. Absolute cephalic size (CS): 591 µm [418, 946]. Cephalic length vs. maximum width of head capsule (CL/CWb): 1.218 [1.057, 1.490]. Postocular distance vs. cephalic length (PoOc/CL): 0.40 [0.359, 0.444]. Scape length vs. absolute cephalic size (SL/CS): 0.676 [0.519, 0.866]. Eye length vs. absolute cephalic size (EL/CS): 0.260 [0.193, 0.317]. Petiole width vs. absolute cephalic size (PEW/CS): 0.431 [0.330, 0.522]. Postpetiole width vs. absolute cephalic size (PPW/CS): 0.496 [0.361, 0.585]. Petiolar node height vs. absolute cephalic size (PEW/CS): 0.250 [0.185, 0.311]. *Nesomyrmex angulatus* (Mayr, 1862) and ca. four undescribed species belong to this group in the Malagasy zoogeographical region.

#### *hafahafa* species-group

Pronotal spines present. Anterodorsal spines on petiolar node present. Propodeal spines long and acute. Vertex ground sculpture areolate. Vertex main sculpture rugulose. metanotal depression absent. Median clypeal notch present. Median clypeal notch shape/depth: 15–31 µm. Antennomere count: 12. Absolute cephalic size (CS): 1059 µm [930, 1200]. Cephalic length vs. maximum width of head capsule (CL/CWb): 1.074 [1.0, 1.143]. Postocular distance vs. cephalic length (PoOc/CL): 0.378 [0.342, 0.403]. Scape length vs. absolute cephalic size (SL/CS): 0.890 [0.835, 0.984]. Eye length vs. absolute cephalic size (EL/CS): 0.232 [0.210, 0.264]. Petiole width vs. absolute cephalic size (PEW/CS): 0.267 [0.203, 0.353]. Postpetiole width vs. absolute cephalic size (PPW/CS): 0.523 [0.430, 0.586]. Petiolar node height vs. absolute cephalic size (PEW/CS): 0.142 [0.107, 0.186]. Four species, *Nesomyrmex capricornis* sp. n., *Nesomyrmex hafahafa* sp. n., *Nesomyrmex medusus* sp. n. and *Nesomyrmex spinosus* sp. n. are known to constitute this species group in Madagascar.

#### *madecassus* species-group

Pronotal spines absent. Anterodorsal spines on petiolar node absent. Propodeal spines short, lamelliform to absent. Vertex ground sculpture smooth. Vertex main sculpture not defined. Metanotal depression present. Median clypeal notch present or absent. Median clypeal notch shape/depth 0–15 µm. Antennomere count: 12. Absolute cephalic size (CS): 571 µm [405, 785]. Cephalic length vs. maximum width of head capsule (CL/CWb): 1.231 [1.092, 1.567]. Postocular distance vs. cephalic length (PoOc/CL): 0.479 [0.407, 0.544]. Scape length vs. absolute cephalic size (SL/CS): 0.718 [0.492, 0.831]. Eye length vs. absolute cephalic size (EL/CS): 0.249 [0.1934, 0.279]. Petiole width vs. absolute cephalic size (PEW/CS): 0.217 [0.181, 0.256]. Postpetiole width vs. absolute cephalic size (PPW/CS): 0.331 [0.243, 0.398]. Petiolar node height vs. absolute cephalic size (PEW/CS): 0.122 [0.072, 0.158]. *Nesomyrmex madecassus* (Forel, 1892) and ca. seven other taxa from the Malagasy zoogeographical region will be revised in the forthcoming revisionary work.

#### *sikorai* species-group

Pronotal spines present or absent. Anterodorsal spines on petiolar node absent. Propodeal spines short to long and acute. Vertex ground sculpture not defined. Vertex main sculpture areolate. Metanotal depression present. Median clypeal notch present or absent. Median clypeal notch shape/depth 0–15 µm. Antennomere count: 12. Absolute cephalic size (CS): 750 µm [634, 890]. Cephalic length vs. maximum width of head capsule (CL/CWb): 1.218 [1.075, 1.382]. Postocular distance vs. cephalic length (PoOc/CL): 0.461 [0.411, 0.511]. Scape length vs. absolute cephalic size (SL/CS): 0.816 [0.761, 0.872]. Eye length vs. absolute cephalic size (EL/CS): 0.232 [0.201, 0.284]. Petiole width vs. absolute cephalic size (PEW/CS): 0.243 [0.206, 0.326]. Postpetiole width vs. absolute cephalic size (PPW/CS): 0.359 [0.306, 0.426]. Petiolar node height vs. absolute cephalic size (PEW/CS): 0.175 [0.149, 0.205]. *Nesomyrmex sikorai* (Emery, 1896), *Nesomyrmex retusispinosus* (Forel, 1892) plus ca. ten more Malagasy species will be revised in a forthcoming revisionary work.

### II. Species delimitation

#### Multivariate Analyses of Numeric Morphology

**Figure 12.**
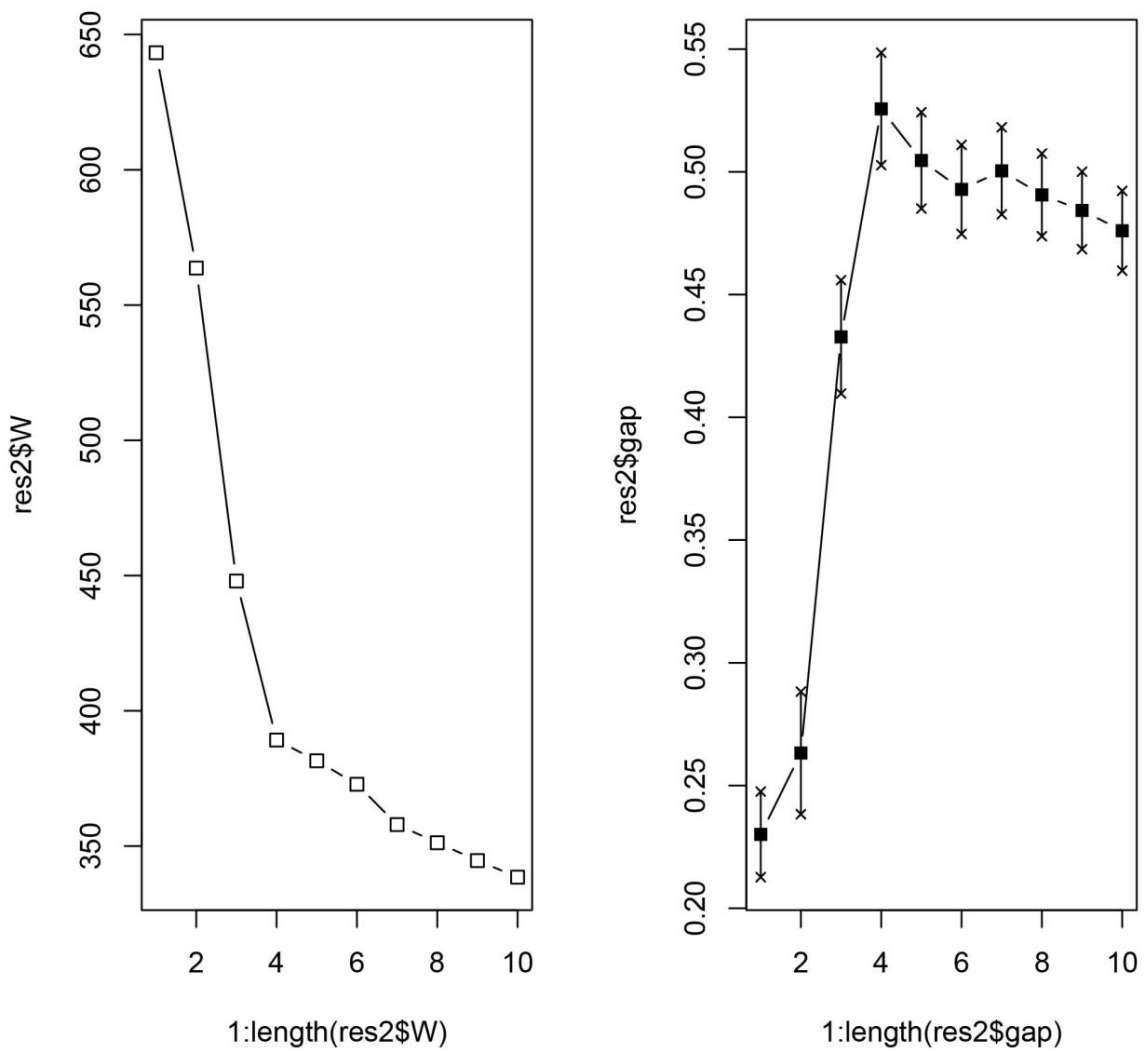
Gap statistic for dataset of *hafahafa* species-group. Four-cluster solution is highly supported by the elbow at 4 components by the dispersion curve (left) and by the peak at cluster number four by the gap curve (right). Number of clusters in the data (X axis), the total within-cluster dispersion for each evaluated partition (Y axis for the left plot) and the vector of length Kmax giving the Gap statistic for each evaluated partition (Y axix for the right plot) is illustrated.

**Figure 13.**
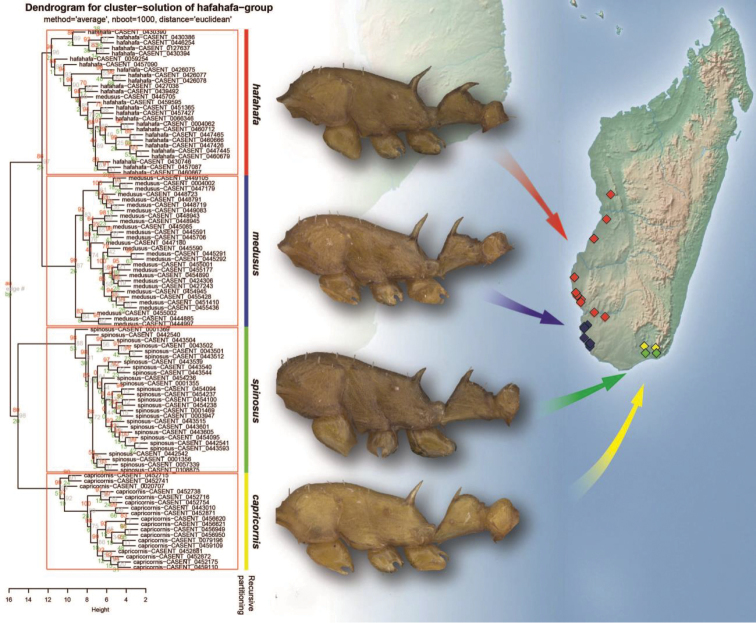
Dendrogram for NC-clustering scores with AU/BP values (%), classification of objects based on recursive partitioning with mesosomal profile of four species of *hafahafa* species-group is mapped on distributional map of Madagascar. Abbreviations: AU = approximately unbiased P-value, BP = bootstrap probabilities before statistical adjustments. Rectangles show the final species hypothesis. Color codes: *Nesomyrmex capricornis* sp. n. (yellow), *Nesomyrmex hafahafa* sp. n. (red), *Nesomyrmex medusus* sp. n. (blue), *Nesomyrmex spinosus* sp. n. (green).

**Figure 14.**
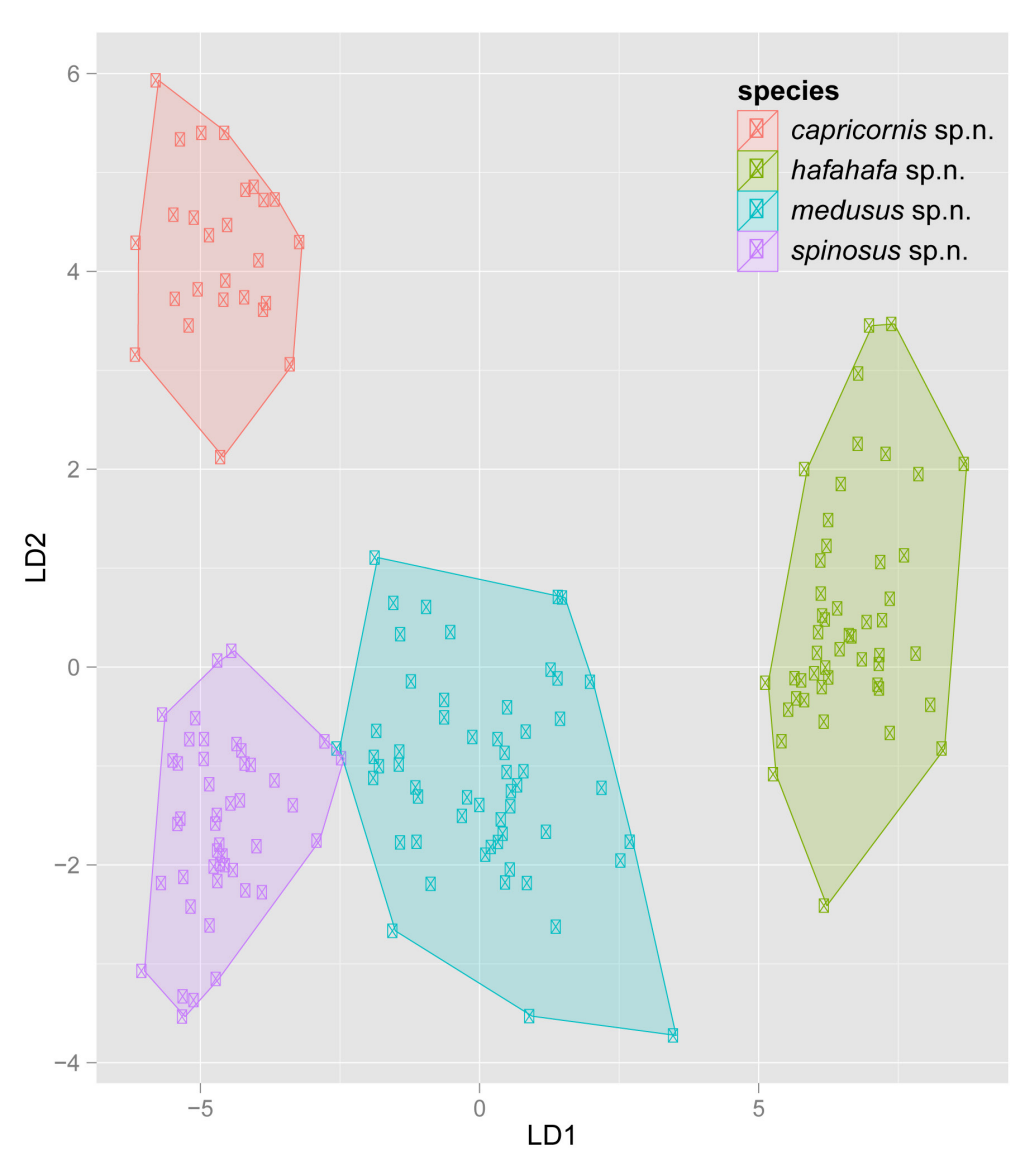
Scatterplot of discriminant scores DL1 and LD2 for *Nesomyrmex capricornis* sp. n. (red), *Nesomyrmex hafahafa* sp. n. (green), *Nesomyrmex medusus* sp. n. (blue), *Nesomyrmex spinosus* sp. n. (lilac) is illustrated. Convex hull graphically displays boundaries between sets of points forming different clusters. Classification functions for LD1 and LD2 are given in the text.

Four clusters were revealed by gap statistic (Fig. 12) to be the most parsimonious solution corroborating the evaluation of the NC-clustering dendrogram (Fig. 13). The grouping hypotheses generated by hypothesis-free exploratory analyses is confirmed by Linear Discriminant Analysis (LDA) with 99.4% classification success. This pattern is also supported by the examination of external morphological traits (e.g. shape of petiolar node, length and deviation of anterodorsal spines on petiolar node), hence the four clusters can be defined as morphospecies based on descriptive morphology. The distinctive morphology of these species permits considerable character reduction, so that the four taxa can be separated based on the combination of four continuous morphometric traits (FRS, NSTI, PSTI and SPST see Table 3) with 99.4% classification success (Fig. 14). Synopses of species were defined based on multivariate analyses of morphological traits: *Nesomyrmex capricornis* sp. n., *Nesomyrmex hafahafa* sp. n., *Nesomyrmex medusus* sp. n., *Nesomyrmex spinosus* sp. n.

**Table 3.** Discriminant scores for each taxon calculated based on classification functions for discriminant roots LD1 and LD2. Scores calculated by classification functions are provided in the following order: mean, ±SD, and minimum, maximum values are given, the latter two in parentheses.

*Nesomyrmex hafahafa* sp. n. (n = 48)	LD1= 6.090±0.76 [4.650, 8.013]
	LD2= 0.547±1.17 [-2.401, 3.491]
*Nesomyrmex medusus* sp. n. (n = 56)	LD1= 0.063±1.27 [-2.299, 3.247]
	LD2= -1.089±1.02 [-3.750, 1.150]
*Nesomyrmex spinosus* sp. n. (n = 46)	LD1= -4.445±0.68 [-5.626, -2.443]
	LD2= -1.623±0.87 [-3.506, 0.170]
*Nesomyrmex capricornis* sp. n. (n = 27)	LD1= -4.373±0.75 [-5.830, -3.065]
	LD2= 4.249±0.84 [2.146, 5.950]

Coefficients of linear discriminants of LD1 and LD2 help to place every additional sample in the discriminant space illustrated in Fig. 14. These placements were calculated using the four most discriminative characters. The morphometric data are in micrometer. Classification functions based on linear discriminants LD1 and LD2 are as follows:

LD1= -(0.0324×PEL)+(0.0121×SPST)-(0.0023×PSTI)+(0.0281×NSTI) +1.6

LD2= +(0.0336×PEL)+(0.0258×SPST)-(0.0328×PSTI)+(0.0049×NSTI)-2.9

Discriminant scores (LD1, LD2) obtained here can either be compared to the values given in Table 3, or can also be used as coordinates in Fig. 14, if relevant scores are fitted on axes LD1 and LD2, and the position of every new sample can be readily identified visually.

Though all species defined in this revisionary work proved to be highly separable via descriptive morphology, or by using simple indices, the application of classification functions LD1 and LD2 provides a foolproof, numeric morphology-based identification tool when decisions based on conventional diagnostic traits fail.

#### Description of the species in the *Nesomyrmex hafahafa* species-group

In this section, four new species of the *Nesomyrmex hafahafa* species-group are described, and a key to these species is provided. Diagnoses are given in the key, the basic statistics of body size ratios are given in Table 4 for each species. The biogeography of the *hafahafa* group is detailed in the discussion. The diagnoses and a key to the four Malagasy *Nesomyrmex* species groups (*angulatus*-group, *hafahafa*-group, *madecassus*-group and *sikorai*-group) defined here are followed by the descriptions of species belonging to the *hafahafa* group.

**Table 4.** Morphometric data of species calculated on individuals. Mean of indices, ±SD are provided in the upper row, minimum and maximum values are given in parentheses in the lower row.

Species:	*Nesomyrmex capricornis* sp. n.	*Nesomyrmex hafahafa* sp. n.	*Nesomyrmex medusus* sp. n.	*Nesomyrmex spinosus* sp. n.
nr. of individulals:	(n = 27)	(n = 48)	(n = 56)	(n = 46)
**CS**	**1024±38**	**1062±41**	**1069±52**	**1021±43**
	[919, 1115]	[974, 1142]	[958, 1189]	[935, 1121]
**CL/CWb**	**1.079±0.020**	**1.038±0.020**	**1.046±0.025**	**1.056±0.024**
	[1.037, 1.111]	[0.993, 1.075]	[0.990, 1.097]	[0.980, 1.113]
**PoOC/CL**	**0.390±0.006**	**0.388±0.010**	**0.391±0.008**	**0.374±0.011**
	[0.381, 0.403]	[0.361, 0.406]	[0.371, 0.413]	[0.342, 0.393]
**FRS/CS**	**0.315±0.007**	**0.316±0.008**	**0.313±0.008**	**0.315±0.009**
	[0.297, 0.326]	[0.289, 0.333]	[0.295, 0.331]	[0.291, 0.335]
**SL/CS**	**0.927±0.012**	**0.895±0.017**	**0.907±0.028**	**0.880±0.016**
	[0.907, 0.948]	[0.861, 0.927]	[0.849, 0.997]	[0.844, 0.919]
**EL/CS**	**0.241±0.011**	**0.230±0.007**	**0.232±0.007**	**0.239±0.008**
	[0.225, 0.267]	[0.212, 0.248]	[0.219, 0.249]	[0.220, 0.265]
**MW/CS**	**0.652±0.012**	**0.657±0.019**	**0.682±0.018**	**0.650±0.014**
	[0.632, 0.685]	[0.631, 0.712]	[0.633, 0.740]	[0.618, 0.679]
**PEW/CS**	**0.265±0.017**	**0.307±0.021**	**0.268±0.011**	**0.237±0.009**
	[0.238, 0.312]	[0.275, 0.357]	[0.246, 0.295]	[0.206, 0.259]
**PPW/CS**	**0.558±0.025**	**0.538±0.022**	**0.543±0.021**	**0.491±0.022**
	[0.516, 0.613]	[0.494, 0.576]	[0.496, 0.585]	[0.435, 0.529]
**SPBA/CS**	**0.260±0.014**	**0.287±0.014**	**0.266±0.018**	**0.212±0.010**
	[0.238, 0.292]	[0.257, 0.311]	[0.234, 0.308]	[0.184, 0.235]
**SPTI/CS**	**0.455±0.039**	**0.543±0.032**	**0.443±0.034**	**0.307±0.027**
	[0.386, 0.569]	[0.463, 0.607]	[0.354, 0.504]	[0.221, 0.361]
**ML/CS**	**1.290±0.026**	**1.266±0.029**	**1.319±0.031**	**1.270±0.023**
	[1.234, 1.335]	[1.201, 1.323]	[1.181, 1.376]	[1.218, 1.313]
**PEL/CS**	**0.506±0.015**	**0.420±0.014**	**0.441±0.018**	**0.435±0.010**
	[0.468, 0.526]	[0.399, 0.453]	[0.392, 0.500]	[0.397, 0.459]
**NOL/CS**	**0.303±0.017**	**0.278±0.015**	**0.290±0.012**	**0.299±0.012**
	[0.258, 0.338]	[0.229, 0.307]	[0.243, 0.319]	[0.265, 0.321]
**PPL/CS**	**0.216±0.007**	**0.202±0.010**	**0.211±0.009**	**0.206±0.011**
	[0.204, 0.228]	[0.181, 0.223]	[0.190, 0.233]	[0.164, 0.231]
**SPST/CS**	**0.397±0.017**	**0.398±0.019**	**0.385±0.019**	**0.300±0.018**
	[0.367, 0.432]	[0.355, 0.427]	[0.333, 0.437]	[0.258, 0.330]
**MPST/CS**	**0.411±0.011**	**0.409±0.013**	**0.400±0.010**	**0.404±0.012**
	[0.386, 0.432]	[0.383, 0.442]	[0.379, 0.426]	[0.370, 0.433]
**PSTI/CS**	**0.658±0.017**	**0.724±0.028**	**0.757±0.020**	**0.677±0.021**
	[0.617, 0.690]	[0.631, 0.776]	[0.711, 0.813]	[0.624, 0.723]
**NSTI/CS**	**0.265±0.035**	**0.514±0.052**	**0.354±0.039**	**0.216±0.018**
	[0.203, 0.364]	[0.473, 0.563]	[0.278, 0.464]	[0.194, 0.276]
**Cdep/CS**	**0.023±0.003**	**0.022±0.003**	**0.022±0.003**	**0.021±0.005**
	[0.018, 0.030]	[0.015, 0.029]	[0.017, 0.029]	[0.015, 0.027]

#### Key to the species of *hafahafa* group

The species of the *Nesomyrmex hafahafa* group differ in body ratios. The following dichotomous identification key for the worker caste was generated based on ratios of morphological features that allow quick identification. Minimum and maximum values for each character is given in parentheses. The reliability of all characters has been tested and calculated classification success was always higher than 95% for each node. Where classification error was detected (i.e. the range of a given trait overlaps between two species) a percentile range 5–95% was also provided in brackets.

**Table:** 

1	Propodeal spine very short (Fig. 15). Spine length vs. absolute cephalic size (SPST/CS): ≤ 0.330 (min. 0.258, max. 0.330)	***spinosus* sp. n.**
–	Propodeal spine longer (Figs 16–18). Spine length vs. absolute cephalic size (SPST/CS): > 0.330 (min. 0.333, max. 0.437)	**2**
2	Bases of anterodorsal petiolar spines enclose a triangular truncate area on the dorsum of petiolar node delineated by a rim (Fig. 16). In dorsal view, anterodorsal petiolar spines distantly surpassing lateral margin of petiole (Fig. 16). Apical distance of the anterodorsal spines on the petiolar node vs. petiole width (NSTI/PEW): > 1.550 (min. 1.531, max. 1.948), [5–95% percentiles: min. 1.563, max. 1.873]	***hafahafa* sp. n.**
–	There is no conspicuous truncate area on the dorsum of petiolar node (Figs 17–18). Apical distance of the anterodorsal spines on the petiolar node vs. petiole width (NSTI/PEW): < 1.550 (min. 0.795, max. 1.575), [5–95% percentiles: min. 0.823, max. 1.549]	**3**
3	In dorsal view, distance between tips of anterodorsal petiolar spines longer than petiole width, spines surpassing lateral margins of petiole (Fig. 17). Apical distance of the anterodorsal spines on the petiolar node vs. petiole width (NSTI/PEW): > 1.090 (min. 1.055, max. 1.575), [5–95% percentiles: min. 1.094, max. 1.549]. Pronotal spines wider; apical distance of pronotal spines vs. absolute cephalic size (PSTI/CS): > 0.700 (min. 0.711, max. 0.813)	***medusus* sp. n.**
–	In dorsal view, distance between tips of anterodorsal petiolar spines shorter than petiole width (Fig. 18). Apical distance of the anterodorsal spines on the petiolar node vs. petiole width (NSTI/PEW): > 1.090 (min. 0.795, max. 1.220), [5–95% percentiles: min. 0.823, max. 1.083]. Apical distance of pronotal spines vs. absolute cephalic size (PSTI/CS): < 0.700 (min. 0.617, max. 0.690)	***capricornis* sp. n.**

**Figures 15–18.**
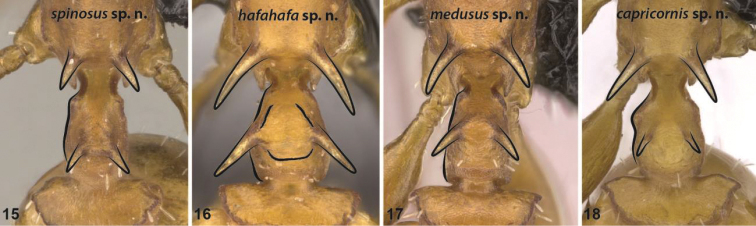
Anterodorsal view of the propodeal spines and anterodorsal spines on the petiolar node of *Nesomyrmex spinosus* sp. n. (**15**), *Nesomyrmex hafahafa* sp. n. (**16**), *Nesomyrmex medusus* sp. n. (**17**), *Nesomyrmex capricornis* sp. n. (**18**). Contour lines of propodeal spines, anterodorsal petiolar spines and the left lateral margin of the petiole are drawn.

#### Nesomyrmex capricornis

Taxon classificationAnimaliaHymenopteraFormicidae

Csősz & Fisher sp. n.

http://zoobank.org/EC84BA51-2D96-4084-AB2B-8B19AF1DEEDC

**Figures 19–21.**
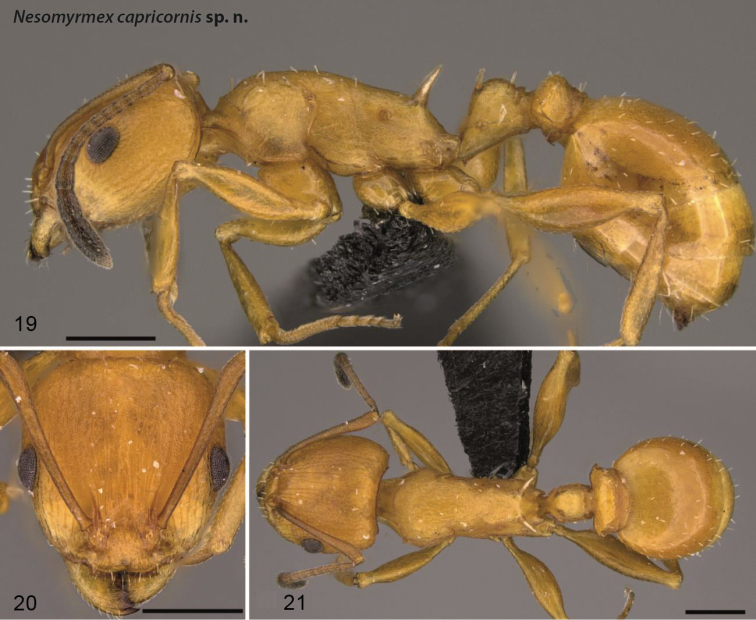
*Nesomyrmex capricornis* sp. n. holotype worker (CASENT0452741). Lateral view of the body (**19**), head of the holotype worker in full-face view (**20**), dorsal view of the body (**21**). Scale 0.5 mm.

Figs 19–21 , Table 4

##### Type material investigated.

**Holotype worker.** CASENT0452741, collection code: BLF05245; MADGAGASCAR: Prov. Toliara, Forêt Mahavelo, Isantoria Riv., 5.2 km 44°NE Ifotaka, 24°46'S, 46°09'E [-24.75833N, 46.15717E], 110 m, 28.iii.2002 Fisher et al. (CAS);

**Paratypes.** Ten workers, a single gyne and two males with the same label data with the holotype under CASENT codes: CASENT0452715, “5245”, (1w, CAS); CASENT0452716, “5245”, (1w, CAS); CASENT0452717, “5245”, (1w, CAS); CASENT0452720, BLF05245, (1w, CAS); CASENT0452721, BLF05245, (1w, CAS); CASENT0452722, BLF05245, (1w, CAS); CASENT0452725, BLF05245, (1w, CAS); CASENT0452726, BLF05245, (1w, CAS); CASENT0452726, BLF05245, (1w, CAS); CASENT0452727, BLF05245, (1w, CAS); CASENT0452728, BLF05245, (1w, CAS); CASENT0452729, BLF05245, (1w, CAS); CASENT0452730, BLF05245, (1w, CAS); CASENT0452731, BLF05245, (1w, CAS); CASENT0452732, BLF05245, (1w, CAS); CASENT0452733, BLF05245, (1w, CAS); CASENT0452734, BLF05245, (1w, CAS); CASENT0452735, BLF05245, (1w, CAS); CASENT0452736, BLF05245, (1w, CAS); CASENT0452737, BLF05245, (1w, CAS); CASENT0452738, BLF05245, (1w, CAS); CASENT0452739, BLF05245, (1w, CAS); CASENT0452742, BLF05245, (1w, CAS); CASENT0452743, BLF05245, (1w, CAS); CASENT0452744, BLF05245, (1w, CAS); CASENT0452745, BLF05245, (1w, CAS); CASENT0452746, BLF05245, (1w, CAS); CASENT0452747, BLF05245, (1w, CAS); CASENT0452748, BLF05245, (1w, CAS); CASENT0452750, BLF05245, (1w, CAS); CASENT0452751, BLF05245, (1w, CAS); CASENT0452752, BLF05245, (1w, CAS); CASENT0452753, BLF05245, (1w, CAS);

The list of 21 non-type individuals belonging to 14 nest samples of other material investigated is given in Table 1.

##### Diagnosis.

In key.

##### Description of workers.

Body color: yellow. Body color pattern: Body concolorous, only clava darker. Absolute cephalic size: 1024 [919, 1115] µm (n=27). Cephalic length vs. maximum width of head capsule (CL/CWb): 1.079 [1.037, 1.111]. Postocular distance vs. cephalic length (PoOc/CL): 0.390 [0.381, 0.403]. Postocular sides of cranium contour frontal view orientation: converging posteriorly. Postocular sides of cranium contour frontal view shape: broadly convex. Vertex contour line in frontal view shape: straight. Vertex sculpture: main sculpture rugose, ground sculpture areolate. Gena contour line in frontal view shape: convex. Genae contour from anterior view orientation: converging. Gena sculpture: rugo-reticulate with areolate ground sculpture. Concentric carinae laterally surrounding antennal foramen count: absent; present. Eye length vs. absolute cephalic size (EL/CS): 0.241 [0.225, 0.267]. Frontal carina distance vs. absolute cephalic size (FRS/CS): 0.315 [0.297, 0.326]. Longitudinal carinae on median region of frons count: present. Longitudinal carinae on medial region of frons shape: forked. Smooth median region on frons count: absent. Antennomere count: 12. Scape length vs. absolute cephalic size (SL/CS): 0.927 [0.907, 0.948]. Facial area of the scape absolute setal angle: setae absent, pubescence only. Median clypeal notch count: present. Median clypeal notch depth vs. absolute cephalic size (Cdep/CS): 0.023 [0.018, 0.030]. Ground sculpture of submedian area of clypeus: smooth. Median carina of clypeus count: present. Lateral carinae of clypeus count: present. Median anatomical line of propodeal spine angle value to Weber length in lateral view: 65–70°. Spine length vs. absolute cephalic size (SPST/CS): 0.397 [0.367, 0.432]. Minimum spine distance vs. absolute cephalic size (SPBA/CS): 0.260 [0.238, 0.292]. Apical spine distance vs. absolute cephalic size (SPTI/CS): 0.455 [0.386, 0.569]. Propodeal spine shape: straight; slightly bent. Apical distance of pronotal spines vs. absolute cephalic size (PSTI/CS): 0.658 [0.617, 0.690]. Metanotal depression count: absent. Dorsal region of mesosoma sculpture: areolate ground sculpture, superimposed by dispersed rugae. Lateral region of pronotum sculpture: areolate ground sculpture, superimposed by dispersed rugae. Mesopleuron sculpture: areolate ground sculpture, superimposed by dispersed rugae. Metapleuron sculpture: areolate ground sculpture, superimposed by dispersed rugae. Petiole width vs. absolute cephalic size (PEW/CS): 0.265 [0.238, 0.312]. Anterodorsal spines on petiolar node angle of deviation from each other: 60°. Apical distance of anterodorsal spines on petiolar node vs. absolute cephalic size (NSTI/CS): 0.265 [0.203, 0.364]. Frontal profile of petiolar node contour line in lateral view shape: straight; concave. Dorso-caudal petiolar profile contour line in lateral view shape: strongly convex. Dorsal region of petiole sculpture: ground sculpture areolate, main sculpture dispersed rugose; ground sculpture areolate, main sculpture absent. Postpetiole width vs. absolute cephalic size (PPW/CS): 0.558 [0.516, 0.613]. Dorsal region of postpetiole sculpture: ground sculpture areolate, main sculpture absent; ground sculpture areolate, main sculpture dispersed rugose.

##### Etymology.

This species is named for the shape of the anterodorsal spines on the petiolar node, which resemble goat horns.

##### Distribution.

This species is known to occur in small, highly isolated forests (Toliara, Forêt Mahavelo and Parc National d’Andohahela, Forêt de Manantalinjo) in the southern part of Madagascar (Fig. 13).

#### Nesomyrmex hafahafa

Taxon classificationAnimaliaHymenopteraFormicidae

Csősz & Fisher sp. n.

http://zoobank.org/C2249F7A-0FFE-4C76-A2E8-905A4B1EA754

**Figures 22–24.**
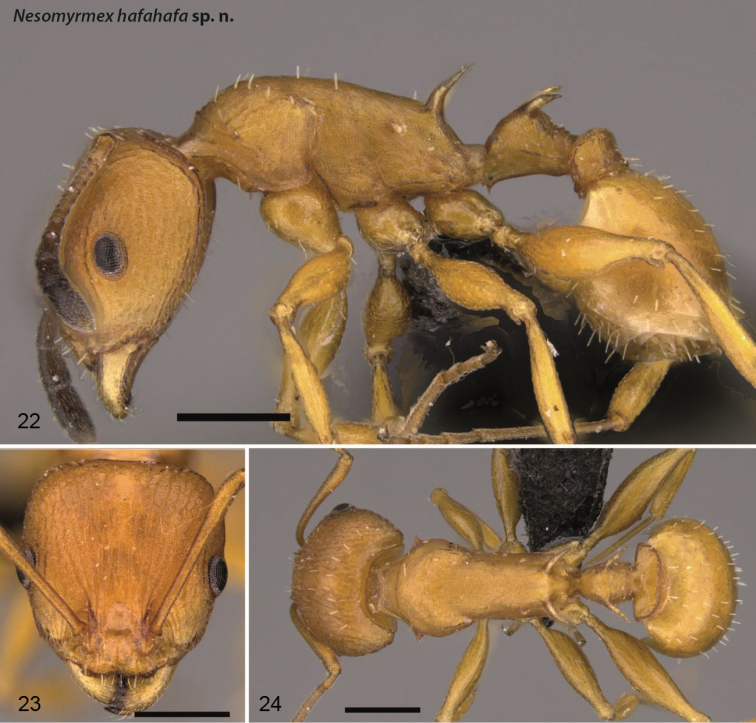
*Nesomyrmex hafahafa* sp. n. holotype worker (CASENT0460666). Lateral view of the body (**22**) head of the holotype worker in full-face view (**23**), dorsal view of the body (**24**). Scale 0.5 mm.

Figs 22–24 , Table 4

##### Etymology.

This Malagasy word “hafahafa” means weird, and refers to the unusual morphology of this species.

##### Type material investigated.

**Holotype worker.** CASENT0460666, collection code: BLF06010; MADG’R: Prov. Toliara, Forêt de Tsinjoriaky, 6.2 km 84° E Tsifota, 22°48'S, 43°25'E [-22.80222N, 43.42067E], 70 m, 6–10.iii.2002 Fisher et al. (CAS)

**Paratypes.** Ten workers, a single gyne and two males with the same label data as the holotype under CASENT codes: CASENT0746771, BLF06010, (2w, CAS); CASENT0460667, BLF06010, (3w, CAS); CASENT0460668, BLF06010, (3w, CAS); CASENT0460669, BLF06010, (1q, CAS); CASENT0451364, “6019”, (2w, CAS); CASENT0451364, “6019”, (2m, CAS);

The list of 44 non-type individuals belonging to 25 nest samples of other material investigated is given in Table 1.

##### Diagnosis.

In key.

##### Description of workers.

Body color: yellow; brown. Body color pattern: body concolorous, only clava darker. Absolute cephalic size: 1062 [974, 1142] µm (n = 48). Cephalic length vs. maximum width of head capsule (CL/CWb): 1.224 [1.193-1.254]. Postocular distance vs. cephalic length (PoOc/CL): 0.388 [0.361, 0.406]. Postocular sides of cranium contour frontal view orientation: converging posteriorly. Postocular sides of cranium contour frontal view shape: broadly convex. Vertex contour line in frontal view shape: straight; slightly concave. Vertex sculpture: main sculpture rugose, ground sculpture areolate. Gena contour line in frontal view shape: feebly convex. Genae contour from anterior view orientation: converging. Gena sculpture: rugo-reticulate with areolate ground sculpture. Concentric carinae laterally surrounding antennal foramen count: present. Eye length vs. absolute cephalic size (EL/CS): 0.230 [0.212, 0.248]. Frontal carina distance vs. absolute cephalic size (FRS/CS): 0.316 [0.289, 0.333]. Longitudinal carinae on median region of frons count: present. Longitudinal carinae on medial region of frons shape: forked. Smooth median region on frons count: absent. Antennomere count: 12. Scape length vs. absolute cephalic size (SL/CS): 0.895 [0.861, 0.927]. Facial area of the scape absolute setal angle: setae absent, pubescence only. Median clypeal notch count: present. Median clypeal notch depth vs. absolute cephalic size (Cdep/CS): 0.022 [0.015, 0.029]. Ground sculpture of submedian area of clypeus: smooth. Median carina of clypeus count: present. Lateral carinae of clypeus count: present. Median anatomical line of propodeal spine angle value to Weber length in lateral view: 55–60°. Spine length vs. absolute cephalic size (SPST/CS): 0.398 [0.355, 0.427]. Minimum spine distance vs. absolute cephalic size (SPBA/CS): 0.287 [0.257, 0.311]. Apical spine distance vs. absolute cephalic size (SPTI/CS): 0.543 [0.463, 0.607]. Propodeal spine shape: strongly bent. Apical distance of pronotal spines vs. absolute cephalic size (PSTI/CS): 0.724 [0.631, 0.776]. Metanotal depression count: absent. Dorsal region of mesosoma sculpture: areolate ground sculpture, superimposed by dispersed rugae. Lateral region of pronotum sculpture: areolate ground sculpture, superimposed by dispersed rugae. Mesopleuron sculpture: areolate ground sculpture superimposed by dispersed rugulae; areolate ground sculpture, superimposed by dispersed rugae. Metapleuron sculpture: areolate ground sculpture, superimposed by dispersed rugae. Petiole width vs. absolute cephalic size (PEW/CS): 0.307 [0.275, 0.357]. Anterodorsal spines on petiolar node angle of deviation from each other: 80°. Apical distance of anterodorsal spines on petiolar node vs. absolute cephalic size (NSTI/CS): 0.514 [0.473, 0.563]. Frontal profile of petiolar node contour line in lateral view shape: convex. Dorso-caudal petiolar profile contour line in lateral view shape: convex. Dorsal region of petiole sculpture: ground sculpture areolate, main sculpture dispersed rugose. Postpetiole width vs. absolute cephalic size (PPW/CS): 0.538 [0.494, 0.576]. Dorsal region of postpetiole sculpture: ground sculpture areolate, main sculpture dispersed rugose.

##### Distribution.

This species is widely distributed along the western forests of Madagascar (Fig. 13) between the 23rd and 20th southern latitudes.

#### Nesomyrmex medusus

Taxon classificationAnimaliaHymenopteraFormicidae

Csősz & Fisher sp. n.

http://zoobank.org/EC3DCF85-8648-4FD2-90D5-113C8FA30099

**Figures 25–27.**
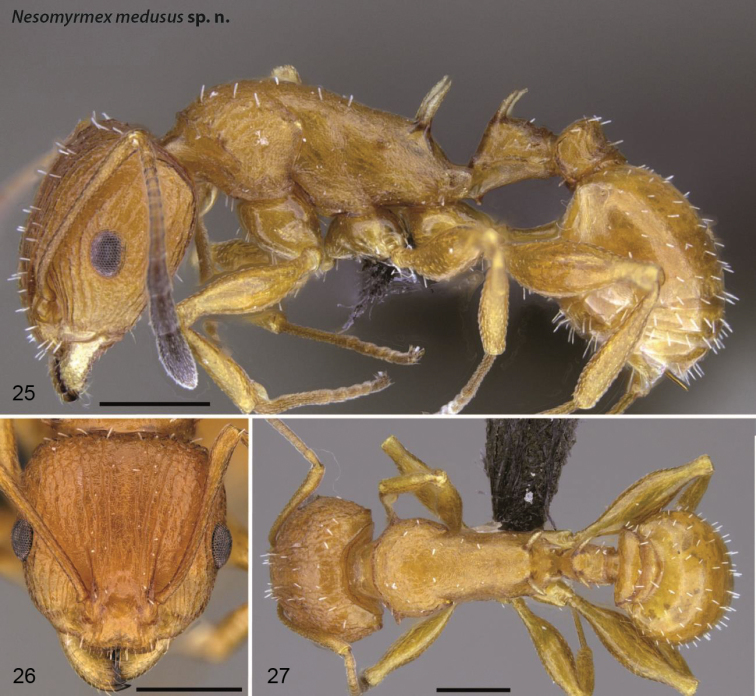
*Nesomyrmex medusus* sp. n. holotype worker (CASENT0455428). Lateral view of the body (**25**), head of the holotype worker in full-face view (**26**), dorsal view of the body (**27**). Scale 0.5 mm.

Figs 25–27 , Table 4

##### Etymology.

The numerous long spines on the dorsal body make the workers reminiscent of Medusa of the Greek mythology who has snakes on her head in place of hair.

##### Type material investigated.

**Holotype worker.** CASENT0455428, collection code: BLF06201; MADGAGASCAR: Prov. Toliara, Parc National de Tsimanampetsotsa, Mitoho Cave, 6.4 km 77° ENE Efoetse, 17.4 km 170°S Beheloka, 24°03'S, 43°46'E [-24.04722 N, 43.75317 E], 65 m, 18–22.iii.2002 Fisher et al. (CAS);

**Paratypes.** Ten workers, a single gyne and two males with the same label data as the holotype under CASENT codes: CASENT0746770, BLF06201, (2w, CAS); CASENT0455429, BLF06201, (3w, CAS); CASENT0455430, BLF06201, (3w, CAS); CASENT0455431, BLF06201, (2w, CAS); CASENT0455432, BLF06201, (2w, CAS); CASENT0455433, BLF06201, (1q, CAS); CASENT0455434, BLF06201, (1w, CAS); CASENT0455435, BLF06201, (1w, CAS); CASENT0455437, BLF06201, (1w, CAS); CASENT0455438, BLF06201, (1w, CAS); CASENT0455439, BLF06201, (1w, CAS); CASENT0455440, BLF06201, (3m, CAS);

The list of 54 non-type individuals belonging to 28 nest samples of other material investigated is given in Table 1.

##### Diagnosis.

In key.

##### Description of workers.

Body color: brown. Body color pattern: body concolorous, only clava darker. Absolute cephalic size: 1069 [958, 1189] µm (n=56). Cephalic length vs. maximum width of head capsule (CL/CWb): 1.046 [0.990, 1.097]. Postocular distance vs. cephalic length (PoOc/CL): 0.391 [0.371, 0.413]. Postocular sides of cranium contour frontal view orientation: converging posteriorly. Postocular sides of cranium contour frontal view shape: broadly convex. Vertex contour line in frontal view shape: straight; slightly concave. Vertex sculpture: main sculpture rugose, ground sculpture areolate. Gena contour line in frontal view shape: feebly convex. Genae contour from anterior view orientation: converging. Gena sculpture: rugo-reticulate with areolate ground sculpture. Concentric carinae laterally surrounding antennal foramen count: present. Eye length vs. absolute cephalic size (EL/CS): 0.232 [0.219, 0.249]. Frontal carina distance vs. absolute cephalic size (FRS/CS): 0.313 [0.295, 0.331]. Longitudinal carinae on median region of frons count: present. Longitudinal carinae on medial region of frons shape: forked. Smooth median region on frons count: absent. Antennomere count: 12. Scape length vs. absolute cephalic size (SL/CS): 0.907 [0.849, 0.997]. Facial area of the scape absolute setal angle: setae absent, pubescence only. Median clypeal notch count: present. Median clypeal notch depth vs. absolute cephalic size (Cdep/CS): 0.022 [0.017, 0.029]. Ground sculpture of submedian area of clypeus: smooth. Median carina of clypeus count: present. Lateral carinae of clypeus count: present. Median anatomical line of propodeal spine angle value to Weber length in lateral view: 65–72°. Spine length vs. absolute cephalic size (SPST/CS): 0.385 [0.333, 0.437]. Minimum spine distance vs. absolute cephalic size (SPBA/CS): 0.266 [0.234, 0.308]. Apical spine distance vs. absolute cephalic size (SPTI/CS): 0.443 [0.354, 0.504]. Propodeal spine shape: straight; slightly bent. Apical distance of pronotal spines vs. absolute cephalic size (PSTI/CS): 0.757 [0.711, 0.813]. Metanotal depression count: absent. Dorsal region of mesosoma sculpture: areolate ground sculpture, superimposed by dispersed rugae. Lateral region of pronotum sculpture: areolate ground sculpture, superimposed by dispersed rugae. Mesopleuron sculpture: areolate ground sculpture, superimposed by dispersed rugae. Metapleuron sculpture: areolate ground sculpture, superimposed by dispersed rugae. Petiole width vs. absolute cephalic size (PEW/CS): 0.268 [0.246, 0.295]. Anterodorsal spines on petiolar node angle of deviation from each other: 70°. Apical distance of anterodorsal spines on petiolar node vs. absolute cephalic size (NSTI/CS): 0.354 [0.278, 0.464]. Frontal profile of petiolar node contour line in lateral view shape: straight. Dorso-caudal petiolar profile contour line in lateral view shape: straight; convex. Dorsal region of petiole sculpture: ground sculpture areolate, main sculpture dispersed rugose; ground sculpture areolate, main sculpture absent. Postpetiole width vs. absolute cephalic size (PPW/CS): 0.543 [0.496, 0.585]. Dorsal region of postpetiole sculpture: ground sculpture areolate, main sculpture absent; ground sculpture areolate, main sculpture dispersed rugose.

##### Distribution.

This species occurs in the south-western forests (Parc National de Tsimanampetsotsa, Forêt de Bemanateza and Mahafaly Plateau) of Madagascar (Fig. 13) between the southern latitudes S 24° and S 24.65°.

#### Nesomyrmex spinosus

Taxon classificationAnimaliaHymenopteraFormicidae

Csősz & Fisher sp. n.

http://zoobank.org/D3643DB1-75EB-415A-9220-9F255A5FCB21

**Figures 28–30.**
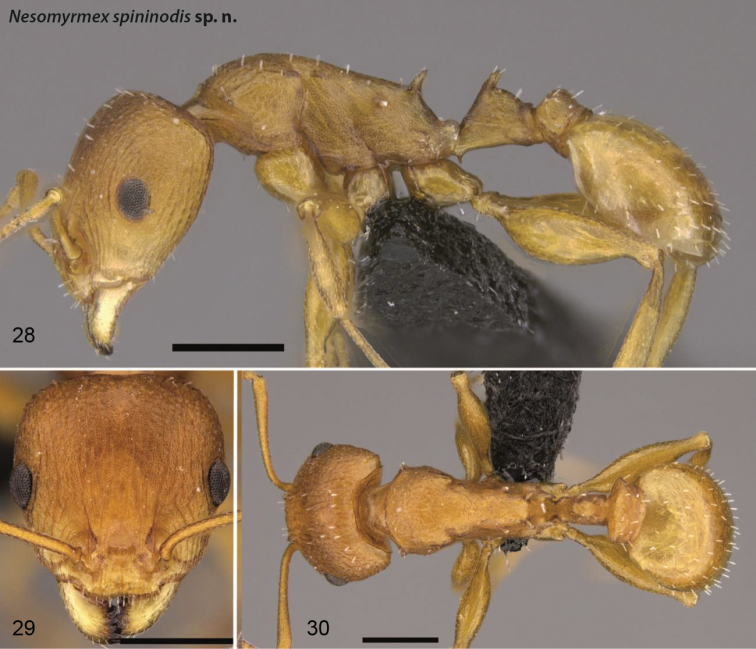
*Nesomyrmex spinosus* sp. n. paratype worker (CASENT0443532). Lateral view of the body (**28**), head of the holotype worker in full-face view (**29**), dorsal view of the body (**30**). Scale 0.5 mm.

Figs 28–30 , Table 4

##### Etymology.

Name “spinosus” refers to the short, strong antero-dorsal spines on the petiolar node.

##### Type material investigated.

**Holotype worker.** CASENT0443515, BLF05489; MADGAGASCAR: Prov. Toliara, Réserve Privé Berenty, Forêt d’Anjapolo, 21.4 km 325° NW Amboasary, 24°56'S, 46°13'E [-24.92972 N, 46.20967 E], 65 m, 7.iii.2002 Fisher et al. (CAS CASENT0443515);

**Paratypes.** 24 workers and three males with the same label data with the holotype under CASENT codes: CASENT0443515, BLF05489, (2w, CAS); CASENT0443516, BLF05489, (3w, CAS); CASENT0443517, BLF05489, (3w, CAS); CASENT0443518, BLF05489, (1w, CAS); CASENT0443519, BLF05489, (1w, CAS); CASENT0443520, BLF05489, (1w, CAS); CASENT0443521, BLF05489, (1w, CAS); CASENT0443522, BLF05489, (1w, CAS); CASENT0443523, BLF05489, (1w, CAS); CASENT0443524, BLF05489, (1w, CAS); CASENT0443525, BLF05489, (1w, CAS); CASENT0443526, BLF05489, (1w, CAS); CASENT0443527, BLF05489, (1w, CAS); CASENT0443530, BLF05489, (1w, CAS); CASENT0443531, BLF05489, (1w, CAS); CASENT0443532, BLF05489, (1w, CAS CASENT0443532); CASENT0443533, BLF05489, (1w, CAS); CASENT0443534, BLF05489, (1w, CAS); CASENT0443535, BLF05489, (1w, CAS); CASENT0443536, BLF05489, (1m, CAS); CASENT0443537, BLF05489, (2m, CAS);

The list of 44 non-type individuals belonging to 26 nest samples of other material investigated is given in Table 1.

##### Diagnosis.

In key.

##### Description of workers.

Body color: brown. Body color pattern: body concolorous, only clava darker. Absolute cephalic size: 1021 [935, 1121] µm (n=46). Cephalic length vs. maximum width of head capsule (CL/CWb): 1.056 [0.980, 1.113]. Postocular distance vs. cephalic length (PoOc/CL): 0.374 [0.342, 0.393]. Postocular sides of cranium contour frontal view orientation: converging posteriorly. Postocular sides of cranium contour frontal view shape: broadly convex. Vertex contour line in frontal view shape: slightly concave. Vertex sculpture: main sculpture rugose, ground sculpture areolate. Gena contour line in frontal view shape: feebly convex. Genae contour from anterior view orientation: converging. Gena sculpture: rugo-reticulate with areolate ground sculpture. Concentric carinae laterally surrounding antennal foramen count: present. Eye length vs. absolute cephalic size (EL/CS): 0.239 [0.220, 0.265]. Frontal carina distance vs. absolute cephalic size (FRS/CS): 0.315 [0.291, 0.335]. Longitudinal carinae on median region of frons count: present. Longitudinal carinae on medial region of frons shape: forked. Smooth median region on frons count: absent. Antennomere count: 12. Scape length vs. absolute cephalic size (SL/CS): 0.880 [0.844, 0.919]. Facial area of the scape absolute setal angle: setae absent, pubescence only. Median clypeal notch count: present. Median clypeal notch depth vs. absolute cephalic size (Cdep/CS): 0.021 [0.015, 0.027]. Ground sculpture of submedian area of clypeus: smooth. Median carina of clypeus count: present. Lateral carinae of clypeus count: present. Median anatomical line of propodeal spine angle value to Weber length in lateral view: 65°. Spine length vs. absolute cephalic size (SPST/CS): 0.300 [0.258, 0.330]. Minimum spine distance vs. absolute cephalic size (SPBA/CS): 0.212 [0.184, 0.235]. Apical spine distance vs. absolute cephalic size (SPTI/CS): 0.307 [0.221, 0.361]. Propodeal spine shape: straight; slightly bent. Apical distance of pronotal spines vs. absolute cephalic size (PSTI/CS): 0.677 [0.624, 0.723]. Metanotal depression count: absent. Dorsal region of mesosoma sculpture: rugose with areolate ground sculpture. Lateral region of pronotum sculpture: areolate ground sculpture, superimposed by dispersed rugae. Mesopleuron sculpture: areolate ground sculpture, superimposed by dispersed rugae. Metapleuron sculpture: areolate ground sculpture, superimposed by dispersed rugae. Petiole width vs. absolute cephalic size (PEW/CS): 0.237 [0.206, 0.259]. Anterodorsal spines on petiolar node angle of deviation from each other: 60°. Apical distance of anterodorsal spines on petiolar node vs. absolute cephalic size (NSTI/CS): 0.216 [0.194, 0.276]. Frontal profile of petiolar node contour line in lateral view shape: straight. Dorso-caudal petiolar profile contour line in lateral view shape: convex. Dorsal region of petiole sculpture: ground sculpture areolate, main sculpture absent; ground sculpture areolate, main sculpture dispersed rugose. Postpetiole width vs. absolute cephalic size (PPW/CS): 0.491 [0.435, 0.529]. Dorsal region of postpetiole sculpture: ground sculpture areolate, main sculpture absent; ground sculpture areolate, main sculpture dispersed rugose.

##### Distribution.

This species is known to occur in small, highly isolated forests (Réserve Privé Berenty, Forêt d’Anjapolo and Parc National d’Andohahela, Forêt d’Ambohibory) in the southern part of Madagascar (Fig. 13).

## Discussion

In this paper we placed the Malagasy *Nesomyrmex* fauna into four species-groups delimited based on morphological features corroborated by morphometric data (see definition and diagnoses of groups). The within-group diversity of one of these new groups, *Nesomyrmex hafahafa* group, was revealed by an enhanced hypothesis-free approach. The exploratory NC-clustering (Seifert et al. 2014) technique was combined with a gap statistic (Tibshirani et al. 2001) in order to address the central problem of taxonomic workflow on estimating the number of optimal clusters (i.e. how many species).

A gap statistic algorithm (function ‘*gap*’) implemented in the package *clusterGenomics* (Nilsen and Lingjaerde 2013) was employed to determine the optimal number of cluster within data that were transformed into discriminant space by the NC-clustering and recursive partitioning (function ‘*part*’) assigned observations (i.e. specimens, or samples) into partitions. Gap statistic is a global method, determines the number of clusters based on gap criterion described by Tibshirani et al. (2001), while recursive partitioning searches for sub-clusters by running ‘gap’ recursively (Nilsen et al. 2013).

Our research demonstrates that combination of NC-clustering with gap statistics and recursive partitioning algorithms performs well in distinguishing partitions in the present data based on morphological distances among nest sample means. Four-cluster hypothesis was returned by both gap statistic (Fig. 12) and recursive partitioning (Fig. 14) as the most parsimonious solution for the diversity of the *hafahafa*-group. This classification was confirmed by multiple lines of evidence. The error rate between the exploratory procedure and the results of the confirmatory Linear Discriminant Analysis was 0.6%. Moreover the pattern recognized by the exploratory process was also corroborated by both the examination of diagnostic morphological traits (e.g. shape of petiolar node, length and deviation of anterodorsal spines on petiolar node) and the known biogeographic patterns (Fig. 14).

We highlight the importance and advantages of the combination of NC-clustering with algorithms to statistically infer gaps and create array of clusters. This protocol also has the potential at accelerate and improve taxonomic decision making process considerably by enabling taxonomists to objectively interpret results based on quantitative morphometric data even in a largely underexplored or poorly understood group such as the Malagasy genus *Nesomyrmex*.

Combination of these approaches allows researchers to recognize cryptic species, but also prevent users from inferring overly diverse pattern in the data. A taxonomist without long-term training in a given group can evaluate new specimens and potential new species by repeating the analysis with measurements from new specimens. This method is best included with an integrated approach that includes conventional morphological characters, biogeography, ecology or molecular data.

## Supplementary Material

XML Treatment for Nesomyrmex capricornis

XML Treatment for Nesomyrmex hafahafa

XML Treatment for Nesomyrmex medusus

XML Treatment for Nesomyrmex spinosus
